# Clinical significance of stratifying prostate cancer patients through specific circulating genes

**DOI:** 10.1002/1878-0261.13805

**Published:** 2025-01-22

**Authors:** Seta Derderian, Edouard Jarry, Arynne Santos, Quentin Vesval, Lucie Hamel, Rafael Sanchez‐Salas, Alexis Rompré‐Brodeur, Wassim Kassouf, Raghu Rajan, Fadi Brimo, Marie Duclos, Armen Aprikian, Simone Chevalier

**Affiliations:** ^1^ Urologic Oncology Research Group, Cancer Research Program Research Institute of the McGill University Health Center Montreal Canada; ^2^ Department of Surgery (Urology Division) McGill University Montreal Canada; ^3^ Department of Urology Centre Hospitalier Régional et Universitaire de Lille France; ^4^ Department of Urology Centre Hospitalier Régional et Universitaire de Rennes France; ^5^ Department of Oncology McGill University Montreal Canada; ^6^ Department of Pathology McGill University Montreal Canada; ^7^ Department of Radiation Oncology McGill University Montreal Canada; ^8^ Department of Medicine McGill University Montreal Canada

**Keywords:** biomarker, drug target, liquid biopsy, neuroendocrine, prostate cancer, stem

## Abstract

Patient stratification remains a challenge for optimal treatment of prostate cancer (PCa). This clinical heterogeneity implies intra‐tumoural heterogeneity, with different prostate epithelial cell subtypes not all targeted by current treatments. We reported that such cell subtypes are traceable in liquid biopsies through representative transcripts. Expanding on this concept, we included 57 genes representing cell subtypes, drug targets and relevant to resistance as non‐invasive biomarkers for stratification. This panel was tested by RT‐qPCR (quantitative reverse transcription polymerase chain reaction) in blood of controls and different categories of PCa patients. Overall, circulating transcripts showed predictive value throughout the disease. Those with aggressive pathological features such as intra‐ductal carcinoma at diagnosis showed more genes over‐expressed. In metastatic patients, signatures of subtypes or resistance were associated with treatments, progression‐free survival and overall survival. Altogether, testing markers of cell diversity, an intrinsic feature of tumours, and drug targets via liquid biopsies represents a valuable means to stratify patients and predict responses to current or new therapeutic modalities. Over‐expressed drug target genes suggest potential benefit from targeted treatments, justifying new clinical trials to offer patient‐tailored strategies to eventually impact on PCa mortality.

## Graphical abstract

We tested a specific panel of genes representative of luminal, neuroendocrine and stem‐like cells in the blood of prostate cancer patients, showing predictive value from diagnosis to late stages of disease. This approach allows monitoring of treatment responses and outcomes at specific time points in trajectories. Administering existing drugs tailored to patients' profiles may open new therapeutic avenues for prostate cancer.

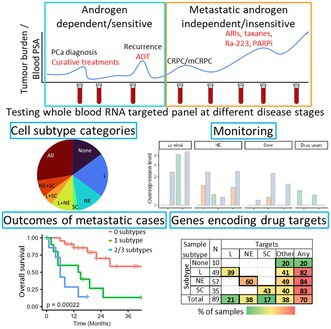

**Abbreviations**
ADT: androgen deprivation therapy
AR: androgen receptor
ARI: androgen receptor inhibitor
CAPRA‐S: Cancer of the Prostate Risk Assessment Score (surgical)
cfDNA: cell‐free DNA
CTC: circulating tumour cell
EMT: epithelial to mesenchymal transition
EV: extracellular vesicle
IDC: intra‐ductal carcinoma
mCRPC: metastatic castration resistant prostate cancer
mHSPC: metastatic hormone‐sensitive prostate cancer
NE: neuroendocrine
NEPC: neuroendocrine prostate cancer
PCa: prostate cancer
PSA: prostate‐specific antigen
RP: radical prostatectomy
RT: radiation therapy
RT‐qPCR: quantitative reverse transcription polymerase chain reaction
WBC: white blood cell

## 1 Introduction

Prostate cancer (PCa) is the most common cancer in North American men and a major cause of cancer mortality. Among patients diagnosed with presumably organ‐confined disease, 25–35% experience a recurrence after curative therapies, including some patients initially considered at low risk. This clinical heterogeneity demonstrates the current inability to predict cancer aggressiveness with certainty at diagnosis, along with the failure of serum prostate‐specific antigen (PSA) and imaging to detect cancer spread beyond the prostate until the disease is advanced [1]. Recurrent disease is treated by androgen deprivation therapy (ADT), which eventually fails and leads to metastatic castration‐resistant PCa (mCRPC).

Intra‐tumoural heterogeneity contributes to clinical heterogeneity. Prostate tumours are composed of a majority of luminal‐like androgen receptor (AR)‐positive cells interspersed with rare AR‐negative cells having neuroendocrine (NE) or stemness features [2, 3]. ADT and AR inhibitors (ARIs) target only AR‐positive cells that depend on androgens for survival. Resistance is attributed to AR‐dependent and ‐independent mechanisms, including selection for (and differentiation towards) AR‐negative NE and stem‐like phenotypes [3, 4, 5]. Taxanes are also used, showing temporary efficacy, but none of these treatments are curative.

Drugs for alternate promising targets have been tested in clinical trials for mCRPC. Few are implemented in practice despite some demonstrating clinical benefit in subsets of unselected patients. This emphasizes the importance of patient stratification to offer personalized therapies. Unfortunately, stratification based on markers in primary tumours may no longer be relevant after years of treatments. Biopsies of metastases would be ideal for identification of reliable prognostic markers, but this invasive procedure is not commonly part of clinical practice. Liquid biopsies, particularly blood, are attractive non‐invasive alternatives that can be collected longitudinally to provide a global view of molecular changes in each patient.

Blood‐based assays can identify proteins, RNAs, cell‐free (cf) nucleic acids, extracellular vesicles (EVs) and circulating tumour cells (CTCs) [6]. Whole‐blood RNA is useful as it encompasses transcripts released from tumours in CTCs and EVs. Most whole‐blood RNA studies focus on early diagnosis, differentiating between cancer and non‐malignant diseases of the corresponding tissue and early detection of recurrence or metastases. Our recent study on 14 cell‐subtype genes in whole‐blood RNA of mCRPC patients revealed phenotypic diversity in luminal, NE and stemness genes [7]. Furthermore, gene over‐expression significantly correlated with treatments received and further progression, thereby supporting their clinical relevance.

Few groups have addressed circulating RNAs as predictors of therapeutic response in PCa. Blood RNA‐sequencing studies identified immune signatures relevant to immunotherapy, targeted therapy and chemotherapy response [8, 9, 10, 11]. These studies did not identify cancer‐ or prostate‐specific genes, which were not detected due to their low expression. In RT‐qPCR studies, progression on taxanes was related to markers of luminal‐like cells, while progression on ARIs involved *AR*, constitutively active *AR* variants (mainly *ARV7*), AR target genes and, as we reported, NE and stemness genes [7, 12, 13, 14, 15, 16]. Altogether, the potential of blood RNA to predict treatment response has been minimally explored, especially in the context of novel therapies.

The present investigation stems from our previous report on cell‐subtype genes in blood of mCRPC patients. We aimed to further investigate the clinical relevance of an expanded gene panel, including not only additional cell‐subtype‐specific genes but also genes reflecting ARI/taxane resistance or encoding alternate therapeutic targets tested in PCa clinical trials but not approved for relevant indications. We demonstrate the predictive value of circulating genes in patients at different stages of disease and identify changes in gene patterns in patients with several blood collections. Accordingly, this approach may be meaningful for identifying subsets of patients for whom more specific tailored therapies may be considered.

## 2 Materials and methods

### 2.1 Patients and controls

This study was approved by the Ethics Review Board of the McGill University Health Center (MP‐37‐2017‐3158 and MP‐37‐2017‐3189 for project and bank, respectively). The study methodologies conformed to the standards set by the Declaration of Helsinki. Participants signed informed consent forms to donate blood for research. Blood samples were collected between May 2017 and June 2023 mainly at the McGill University Health Center, with 15% at the Jewish General Hospital. Eighty‐nine blood samples were drawn from 65 new patients: (a) 16 before radical prostatectomy (RP); (b) 26 post‐curative therapies but not metastatic, including 5 patients who were also tested pre‐RP; (c) 28 metastatic (3 hormone‐sensitive (mHSPC) and 25 mCRPC). Blood was also collected from 26 controls with no history of cancer, consisting of 20 males with no prostatic diseases and 6 females. A validation cohort was included, consisting of 37 samples from 32 mCRPC patients. A pseudonymized database was generated from patients' medical records. Baseline characteristics for patients and controls are displayed in Table S1.

### 2.2 Blood processing and RNA extraction

Peripheral blood was collected in PAXgene tubes (Qiagen, Germantown, MD, USA), incubated at room temperature for 2 h for cell lysis, frozen at −20 °C and stored long term at −80 °C. RNA was extracted using PAXgene RNA extraction kits following Qiagen protocols. RNA integrity and concentration were determined by BioAnalyzer 2100 (Agilent, Millcreek, ON, Canada) and Nanodrop ND‐1000 (Thermo Scientific, Waltham, MA, USA), respectively. RNA was stored at −80 °C and remained high quality over time.

### 2.3 RT‐qPCR assays

Total RNA (500 ng) was reverse‐transcribed using the Maxima H Minus First Strand cDNA Synthesis Kit with dsDNase (Thermo Scientific). A negative control with no template was run in parallel. TaqMan assays were obtained from Thermo Scientific or Integrated DNA Technologies, either as pre‐designed or custom assays. Probe and primer information are available in Table S2. All qPCR reactions were carried out in triplicate using the TaqMan Fast Advanced Master Mix (Thermo Scientific) on the CFX384 TouchTM (Bio‐Rad, Mississauga, ON, Canada) with the following program: UNG incubation 50 °C 2 min, polymerase activation 95 °C 20 s, 45 cycles of denaturation (3 s 95 °C) and annealing/extension (30 s 60 °C).

Human PCa cell lines LNCaP (Research Resource Identifiers (RRID): CVCL_0395), DU145 (RRID: CVCL_0105) and PC‐3 (RRID: CVCL_0035) were from ATCC (Manassas, VA, USA). NCI‐H660 (RRID: CVCL_1576) and 22Rv1 (RRID: CVCL_1045) were generous gifts from Dr A. Zoubeidi (Prostate Centre, Vancouver, BC, Canada) and Dr M. Tremblay (McGill Goodman Cancer Institute, Montreal, QC, Canada), respectively. Cultures were consistently started from frozen cells at low passages within 3 years of purchase to ensure reproducibility of results. Cells were cultured according to ATCC recommendations for no more than 30 passages, with routine mycoplasma testing (MycoFluor Mycoplasma kit; Thermo Scientific). Assay efficiency was quantified for each gene using serial dilutions of a mix of RNA from these five human PCa cell lines or RNA from the NCI‐H660 cell line alone (Table S2).

For experiments on blood RNA, each plate included a negative control (no template in cDNA synthesis) and serial dilutions (RNA mix from cell lines) for each gene and at least one reference gene. Relative normalized gene expression was determined by the 2^−Δ*C*t^ method. Reference genes (*PGK1*, *PPIB*, *RPLP0*) were chosen as previously described [7].

### 2.4 Statistical analyses

Gene over‐expression in blood was defined as either 2.58 standard deviations above the mean expression in controls or the maximum expression detected in controls (whichever was highest). Only male controls were used for cut‐off determination for nine genes showing a difference (*P* < 0.1) from female controls (*AR*, *ENO2*, *EPCAM*, *IGFBP2*, *INSM1*, *MET*, *POU3F2*, *SRC*, *VIM*).

Spearman correlation was used to analyse associations between gene expression with patient's age and blood counts. To analyse the association of individual genes vs. patient's characteristics or treatments, differences were evaluated using chi‐square tests. To identify characteristics/treatments associated with over‐expression of gene sets, we adjusted for repeated measures within individuals using multi‐level mixed‐effects logistic regression, while also adjusting for multiple blood draws for 14 patients. Kaplan–Meier and Cox proportional hazards models were used in survival analyses for disease progression and overall survival. Progression was either clinical (worsening of symptoms), biological (PSA rising in two subsequent measurements) or radiological (new lesion or increased size of existing lesions). Results with *P* < 0.05 were considered significant. All statistical analyses were performed using stata (v16.1, StataCorp LLC, College Station, TX, USA) and r (v 4.3.2, r core team 2023, vienna, austria).

### 2.5 Bioinformatic analyses

Cell‐line RT‐qPCR results were compared to RNA‐sequencing data from the Cancer Cell Line Encyclopedia Expression Atlas [17]. Gene expression data of individual white blood cell (WBC) populations were obtained from the consensus dataset from the Human Protein Atlas (proteinatlas.org) [18]. Gene expression microarray data were accessed through Gene Expression Omnibus for the Stanford (GSE3933), Cambridge (GSE70770) and MSKCC (GSE21032) datasets. For the Stanford dataset, data were normalized as initially reported [7]. For MSKCC and Cambridge datasets, data were normalized using the LIMMA Bioconductor package in r. Publicly available RNA‐sequencing datasets were accessed through Gene Expression Omnibus for red blood cells (GSE108378), platelets (GSE89843) and whole blood (GSE181228). RNA‐sequencing data under controlled access were obtained through dbGaP for SU2C (phs000915.v2.p2) and GDC for TCGA (phs000178.v11.p8). Raw data was processed using the Canadian Centre for Computational Genomics GenPipes RNA‐seq pipeline, with count normalization using the deseq2 Bioconductor packages in r. Gene over‐expression in tumours was defined as 2.58 standard deviations over the mean expression in benign samples from the same dataset; for SU2C, metastases were compared to TCGA benign samples after batch correction using the LIMMA Bioconductor packages in r.

## 3 Results

### 3.1 Selected genes reflect the aggressiveness of PCa

**Fig. 1:**
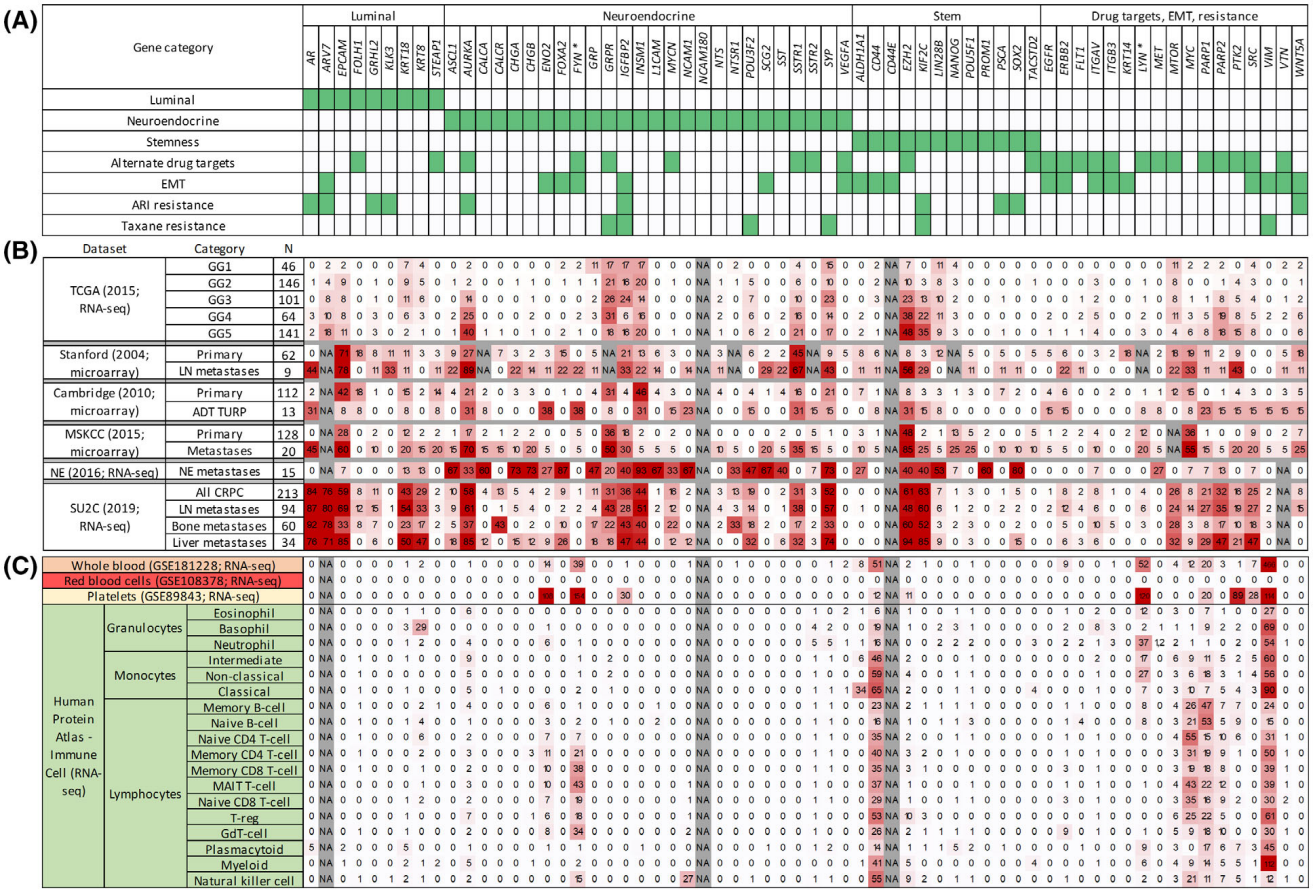
Selection of gene panel and expression in various PCa cohorts, whole blood and components thereof. (A) Our panel of 64 genes is presented by cell subtype, drug targets, EMT or ARI/taxane resistance. (B) Expression of these genes in five prostate cancer (PCa) transcriptomic datasets. Results are presented as the proportion of cases over‐expressing each gene in each category of patients. Categories include RP cases by Gleason grade (GG), lymph node (LN) metastases at initial diagnosis, transurethral resection of the prostate (TURP) of ADT cases and various CRPC metastases, including neuroendocrine PCas (NEPC). For TCGA, Stanford, Cambridge and MSKCC datasets, over‐expression was defined as 2.58 standard deviations over the mean of controls in the same dataset. For the NE dataset, NEPC cases were compared to non‐NE CRPC cases, with a cut‐off of 2.58 standard deviations. In the SU2C dataset, CRPC metastases were compared to TCGA benign samples after normalization, as described in Section 2. The gradient from white to red represents increasing proportion of cases with over‐expression. (C) Expression levels of the same genes in blood datasets. For whole blood, red blood cells and platelets, RNA‐sequencing results are presented as transcripts per million (TPM). Results for white blood cell (WBC) populations are presented in normalized TPM, as described on the Human Protein Atlas. The gradient from white to red represents increasing expression.

Our initial panel included 14 genes representative of prostate luminal, NE and stem‐like cells [7]. We first aimed to better represent intra‐tumoural heterogeneity not only with additional genes of these cell subtypes but also genes accounting for mechanisms of resistance to ARIs and taxanes or encoding targets for existing drugs tested in PCa clinical trials (Fig. 1A). Inclusion and exclusion criteria for expanding this panel are detailed in Table 1. The 64 chosen genes were first validated in PCa tissue transcriptomic datasets representing different categories of patients (Fig. 1B). Most genes were over‐expressed in at least one category of cases, particularly in advanced disease and predominantly in metastases.

**Table 1:** Criteria for gene panel selection. ARI, androgen receptor inhibitors; CTC, circulating tumor cell; EMT, epithelial to mesenchymal transition; mCRPC, metastatic castration‐resistant prostate cancer; NE, neuroendocrine; NEPC, NE prostate cancer; PCa, prostate cancer.

	Source
Inclusion criteria	
Epithelial cell‐subtype‐specific	Systematic literature review on luminal, neuroendocrine and stemness genes in PCa
	Upregulated in NEPC vs. non‐NE CRPC (NEPC dataset)
EMT in PCa	Systematic literature review on EMT signatures in PCa
Targets of most interesting drugs tested in clinical trials for PCa	Systematic review of clinical trial results and literature on drugs/targets (up to 2024)
Resistance to ARIs	Systematic literature review on AR inhibitor resistance, including clinical studies, *in vitro* testing and CTCs in ARI‐treated mCRPC cases
	RNA‐sequencing of diverse mCRPC metastases (ARI‐treated vs. ARI‐naïve cases)
Resistance to taxanes	Systematic literature review on taxane resistance, including clinical and *in vitro* studies
	RNA‐sequencing of diverse mCRPC metastases (taxane‐treated vs. taxane‐naïve cases; SU2C dataset)
Exclusion criteria	
High expression in whole blood RNA	GSE181228 (RNA‐sequencing)
High expression in specific white blood cell population(s)	Human Protein Atlas (RNA‐sequencing)
High expression in red blood cells	GSE108378 (RNA‐sequencing)
High expression in platelets	GSE89843 (RNA‐sequencing)
Difference by age in whole blood of healthy individuals	GSE33828 (microarray)

As mentioned in Table 1, genes with high expression in blood or specific blood cells were excluded. Indeed, 52/64 genes showed negative or minimal expression in blood components (Fig. 1C). The epithelial to mesenchymal transition (EMT) gene *VIM* was removed due to high expression in several WBCs. For certain genes showing expression in blood, we opted for assays targeting cancer‐specific splice variants (Table 1; *NCAM180*, *CD44E*, *LYN56*, *FYN‐B*). Two other exceptions were kept as our previous study showed that they do not correlate with patients WBCs or platelets (*ENO2*, *ALDH1A1*) [7]. At this point, all 63 genes appear suitable and were kept for further validation.

### 3.2 Chosen assays allow for specific and reproducible testing of circulating genes

We next carried out technical and biological validation of the chosen assays. Assays were first tested in five PCa cell lines, showing expression patterns in line with RNA‐sequencing data available from the CCLE (Fig. S1A).

**Fig. 2:**
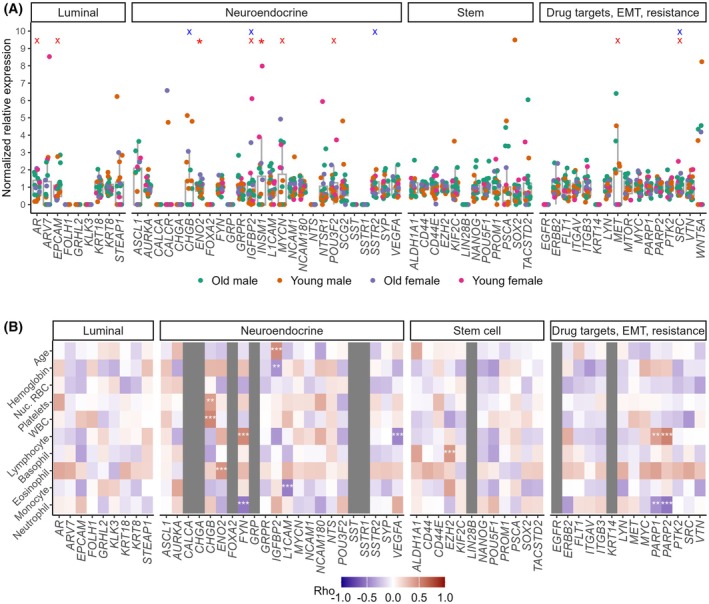
Biological validation of gene panel. (A) Normalized gene expression is presented for healthy controls, grouped as younger vs. older (< vs. > 55 years) and male vs. female, with comparisons by Wilcoxon rank‐sum test; *P* values < 0.05 (*) or < 0.1 (x) are shown in blue and red for differences by age and biological sex, respectively. Whiskers of box plots represent 1.5 times the interquartile range. (B) Heatmap of Spearman correlation results between gene expression in patients' blood vs. age, haemoglobin, platelets and white blood cell counts. Only genes showing consistent results are presented, as described in Fig. S3. Colours indicate Rho values, where red and blue represent positive and negative correlations, respectively. Significant *P*‐values are denoted by ** for *P* < 0.01; *** for *P* < 0.001. EMT, epithelial to mesenchymal transition; Nuc RBC, nucleated red blood cells; WBC, white blood cell.

To determine cut‐offs for gene over‐expression in patients, we tested all genes in the blood of 26 controls (Fig. 2A). Twelve of 63 genes were completely negative in all controls, while the remaining showed low expression levels. No genes correlated with age despite the increased incidence of prostatic diseases in aging men. However, some genes (*EPCAM*, *ENO2*, *INSM1*, *POU3F2*) differed between male and female controls. These sex‐based differences were considered for cut‐off determination for each gene, as described in Section 2.4.

We next demonstrated reproducibility of results. A control sample tested in two separate RNA extractions and two separate cDNA synthesis reactions yields consistent results remaining below thresholds (Fig. S1B). Furthermore, longitudinally collected samples from two controls (15–18 months) showed that gene expression remained below set thresholds and was relatively constant over time (Fig. S2). Testing genes in 10 patients showed reproducible results of over‐expression throughout experiments for 57/63 genes, which were thus kept for further analyses (Fig. S3).

We also verified that there is minimal correlation between genes and patients' age and blood counts (Fig. 2B). The few positive correlations were further analysed and found to be weak and non‐linear (Fig. S4A). Patients' relative blood cell counts are also shown (Fig. S4B). Collectively, these findings confirmed the specificity, reproducibility and suitability of assays to test 57 circulating genes in whole‐blood RNA.

### 3.3 Circulating genes are over‐expressed at all stages of PCa

The 57 gene panel was next tested in a total of 89 blood samples from patients, including cases prior to RP, non‐metastatic HSPC cases and mHSPC or mCRPC patients. Gene over‐expression results are presented in a heatmap (Fig. S5). A total of 44/57 genes were over‐expressed in at least one patient sample, 5/57 showed baseline expression and 9/57 were completely negative in all blood samples tested. Overall, this confirms the heterogeneity of circulating gene patterns between and within categories of patients.

### 3.4 Circulating gene patterns at diagnosis are related to aggressive pathological features and evolve during the course of disease

**Fig. 3:**
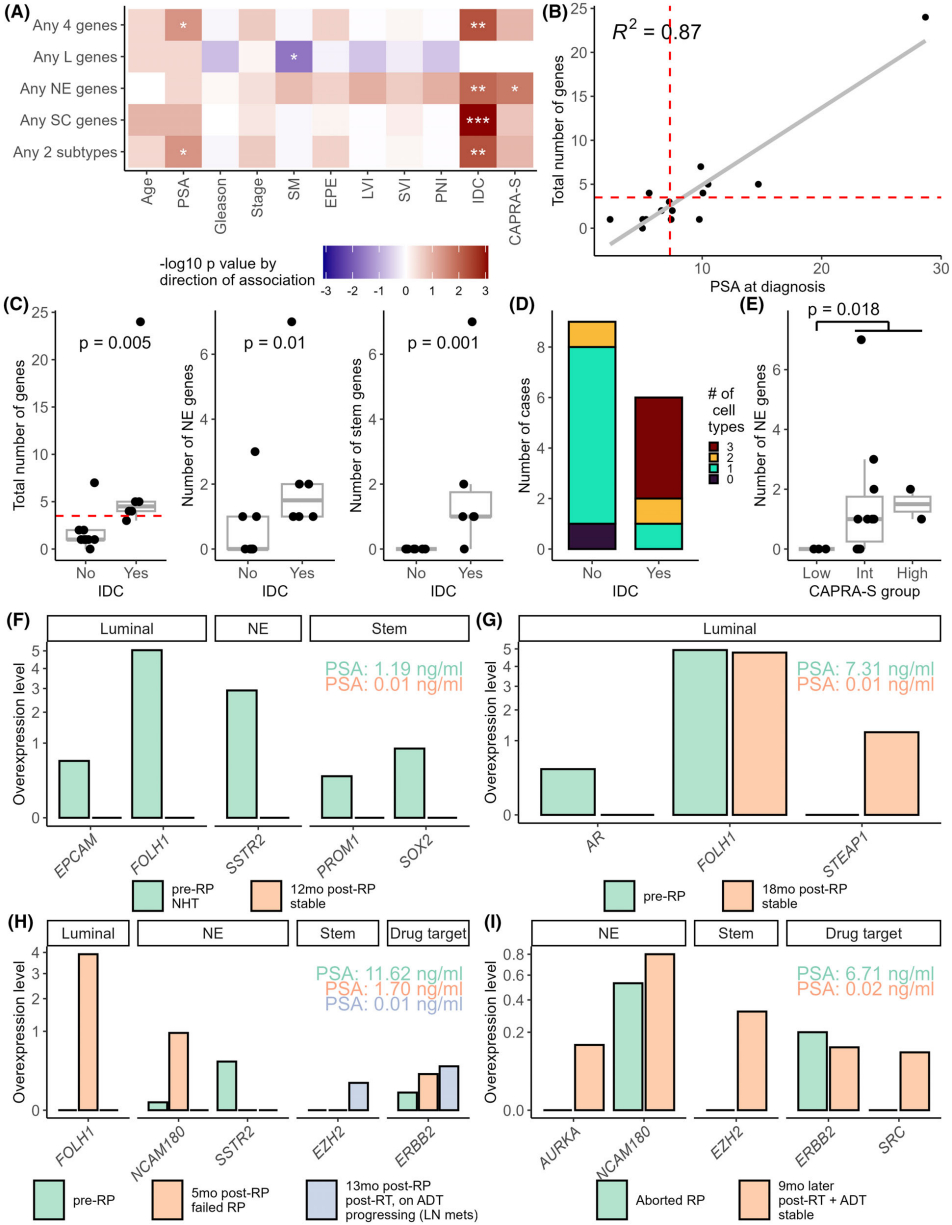
Clinical relevance of circulating genes over‐expressed in patients prior to RP. Analyses were carried out in blood of patients prior to (A–E) or post RP (F–I). (A) Heatmap of χ^2^ results for associations between over‐expression of genes in patients pre‐RP vs. clinical characteristics and pathological features from surgery. Red and blue represent positive and negative correlations, respectively. χ^2^ *P*‐values for significant associations are indicated: * for *P* < 0.05; ** for *P* < 0.01; *** for *P* < 0.001. (B) Scatter plot representing the total number of genes over‐expressed vs. blood prostate‐specific antigen (PSA) at diagnosis. The vertical dashed line represents the median PSA in these cases. The horizontal dashed line represents ≥ 4 genes over‐expressed. (C) Box plots representing the number of genes over‐expressed overall or by cell subtype as a function of intraductal carcinoma (IDC; *n* = 9 without IDC, *n* = 6 with IDC). Whiskers of box plots represent 1.5 times the interquartile range. χ^2^ *P*‐values are indicated. The dashed red line indicates the cutoff of any four genes over‐expressed. (D) Stacked bar graphs representing the number of cell subtypes in each case by the presence or absence of IDC. (E) Box plots representing the number of neuroendocrine (NE) genes over‐expressed vs. CAPRA‐S risk groups (*n* = 3 low risk, *n* = 10 intermediate risk and *n* = 2 high risk). Whiskers of box plots represent 1.5 times the interquartile range. χ^2^ *P*‐values are indicated. (F–I) Gene over‐expression in patients with multiple blood collections. Collections are presented chronologically, with details of treatments and progression indicated. PSA values are indicated in the same colour as the respective time point. ADT, androgen deprivation therapy; CAPRA‐S, Cancer of the Prostate Risk Assessment Score (surgical); EPE, extraprostatic extension; IDC, intraductal carcinoma; Int, intermediate; L genes, luminal genes; LN, lymph node; LVI, lymphovascular invasion; NHT, neoadjuvant hormone therapy; PNI, perineural invasion; PSA, prostate‐specific antigen; RT, radiation therapy; SC, stem cell; SM, surgical margin; SVI, seminal vesicle invasion.

Fewer circulating genes were detected in patients prior to RP than in other categories. Only 1/16 patients had no genes over‐expressed. 30/57 genes were over‐expressed in blood of at least one patient, of which four were found in at least 25% of cases (*FOLH1*, *SSTR2*, *L1CAM*, *NCAM180*; Fig. S5). Chi‐squared results revealed significant correlations between patients' clinical and pathological parameters with the number of genes over‐expressed, cell subtype‐specific genes and the number of cell subtypes represented (Fig. 3A). For instance, patients with higher blood PSA at diagnosis were more likely to have four or more genes over‐expressed or any two cell types (Fig. 3A,B). Patients with intra‐ductal carcinoma (IDC) were more likely to have four or more genes over‐expressed overall or at least one NE gene (Fig. 3A,C). These patients were also the only ones over‐expressing stemness genes and genes of all cell subtypes (Fig. 3A,C,D). Finally, patients with a higher risk score (based on CAPRA‐S classification) had more NE genes (Fig. 3A,E).

A subset of these cases also had blood collections post‐RP, for which results are presented in Fig. 3F–I. The patient presented in Fig. 3F had a high number of genes over‐expressed pre‐RP, representing the three cell subtypes, but no genes at 12 months post‐RP. In contrast, the patient in Fig. 3G has two luminal genes pre‐RP, of which the prostate‐specific gene *FOLH1* persists at 18 months post‐RP, along with another potentially targetable luminal gene, *STEAP1*. Neither of these cases showed signs of clinical progression post‐RP at last follow‐up. We also show a patient who failed RP in Fig. 3H. He had three over‐expressed genes pre‐RP, two of which remained at 5 months post‐RP with persisting blood PSA when *FOLH1* was also over‐expressed. A shift in gene profile was also noted upon radiation therapy (RT)/ADT at 13 months, with no remaining luminal or NE genes and continued over‐expression of the drug target *ERBB2*, and the presence of the stemness gene *EZH2*. Interestingly, one patient whose RP was aborted for anatomical issues had sustained over‐expression of two genes present at diagnosis and gained three additional genes at 9 months post‐RT/ADT while being stable with a low PSA (Fig. 3I).

Altogether, gene over‐expression in blood is common at diagnosis and is related to worse clinical and pathological features. Further testing post‐RP revealed changes with treatments traceable within months and reflecting outcomes.

### 3.5 Circulating genes in recurrent non‐metastatic patients

Analyses were more restrictive in recurrent non‐metastatic patients. No patients were actively progressing, and the cohort was heterogeneous. While the number of over‐expressed genes did not differ between ADT‐naïve and ADT‐treated cases, the patients with the most genes over‐expressed were all ADT‐naïve (Fig. S6A). However, the stemness gene *EZH2* was more commonly over‐expressed in patients on ADT (Fig. S6B). Furthermore, *EZH2* and the NE gene *IGFBP2* were more common in patients who underwent radiotherapy (Fig. S6B).

### 3.6 Circulating genes in metastatic cases correlate with clinical characteristics and treatments

**Fig. 4:**
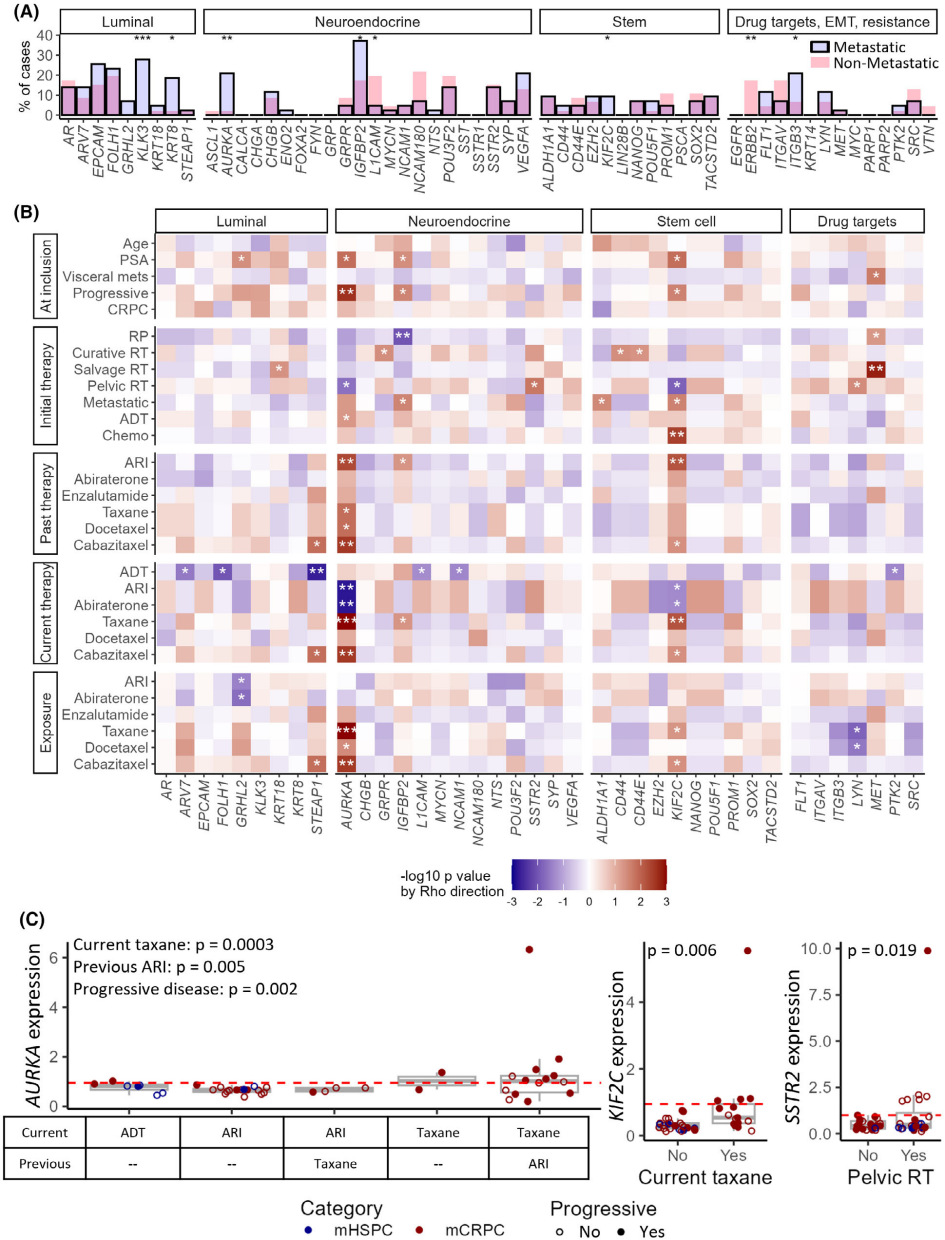
Circulating genes correlate with clinical parameters and treatments in metastatic patients. (A) Bar graph representing the proportion of cases over‐expressing each gene for metastatic (blue bars) and non‐metastatic cases (pink bars). Significant χ^2^ *P*‐values are denoted by * for *P* < 0.05; ** for *P* < 0.01; *** for *P* < 0.001. (B) Heatmap of χ^2^ results for associations between over‐expression of genes in metastatic patients and clinical characteristics or treatments. Red and blue represent positive and negative correlations, respectively. Significant associations are indicated: * for *P* < 0.05; ** for *P* < 0.01; *** for *P* < 0.001. (C) Box plots representing expression of *AURKA*, *KIF2C* and *SSTR2* (from left to right) vs. therapeutic interventions and progressive disease. Cases with metastatic hormone‐sensitive prostate cancer (mHSPC) and castration‐resistant prostate cancer (mCRPC) are presented in blue and red, respectively; cases with progressive disease are represented as filled red/blue circles, while patients not actively progressing are shown as empty circles. *P* values for χ^2^ results are indicated. Whiskers of box plots represent 1.5 times the interquartile range. The dashed red lines indicate the over‐expression threshold. ADT, androgen deprivation therapy; ARI, androgen receptor inhibitor; EMT, epithelial to mesenchymal transition; mCRPC, metastatic castration resistant prostate cancer; mHSPC, metastatic hormone‐sensitive prostate cancer; PSA, prostate‐specific antigen; RP, radical prostatectomy; RT, radiation therapy.

The most meaningful findings were obtained in metastatic patients, where 41/57 genes were over‐expressed in at least one sample. Given that these genes were mostly relevant to advanced and/or metastatic disease (Fig. 1), we compared their over‐expression in blood of metastatic vs. non‐metastatic patients. Chi‐squared analyses showed that six genes were over‐expressed more often in metastatic cases, including some seen strictly in mCRPC (*KLK3*, *KIF2C*), and two in non‐metastatic cases (*ERBB2*, *L1CAM*; Fig. 4A). Interestingly, mixed‐effects logistic regression models revealed that metastatic cases were more likely to over‐express luminal genes and genes related to treatment resistance compared to non‐metastatic patients (Table 2).

**Table 2:** Comparison of circulating genes in metastatic vs. non‐metastatic patients. Mixed‐effects logistic regression for gene signatures in metastatic vs. non‐metastatic patients. Significant results are presented in bold. ARI, androgen receptor inhibitor; CI, confidence interval; EMT, epithelial to mesenchymal transition; HR, hazard ratio; SC, stem cell.

Metastatic vs. non‐metastatic	HR	95% CI
Luminal	**2.79**	**1.30–5.98**
Luminal (prostate‐specific)	**2.50**	**1.09–5.71**
Luminal/epithelial	**3.59**	**1.11–11.55**
Neuroendocrine	1.01	0.53–1.94
Stem cell	0.91	0.36–2.28
EMT	1.30	0.61–2.77
SC + EMT	1.08	0.35–3.35
ARI resistance	**3.29**	**1.57–6.93**
Taxane resistance	2.00	0.85–4.72
ARI and taxane resistance	**5.44**	**1.56–6.93**

In metastatic cases, various genes were related to clinical features and treatments (Fig. 4B), with examples shown in Fig. 4C. The NE gene *AURKA* was over‐represented in patients on current taxane treatment (irrespective of ARI status), especially in those progressing at inclusion, and in patients who have progressed on various treatments (Fig. 4B,C). The stemness gene *KIF2C* was elevated in cases treated with taxanes at diagnosis, those who progressed on ARIs, and in cases currently on taxanes (Fig. 4B,C). The NE gene *SSTR2* was exclusively over‐expressed in patients who received pelvic radiotherapy (Fig. 4B,C). Interestingly, no genes were significantly over‐represented in CRPC cases compared to HSPC (Fig. 4B). Moreover, over‐expression of *AURKA* and *IGFBP2* was associated with progression (Table S3). *AURKA* and the luminal genes *GRHL2* and *STEAP1* were associated with an increased risk of death (Table S3).

### 3.7 Circulating genes in metastatic cases tested over time reflect progression and changes in treatments

**Fig. 5:**
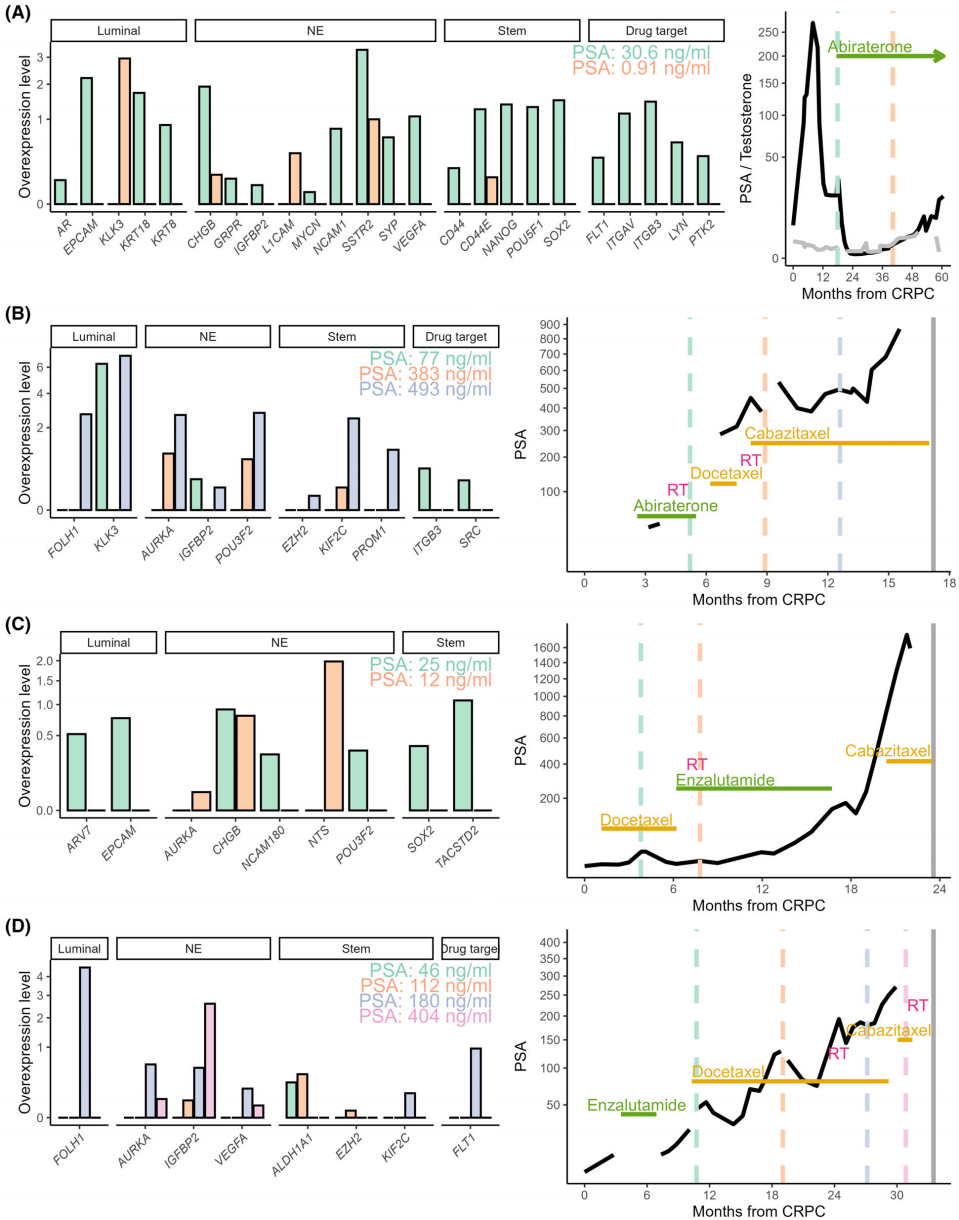
Monitoring of circulating genes in mCRPC patients over time. (A–D) Gene over‐expression is presented for metastatic castration resistant prostate cancer (mCRPC) patients with multiple blood collections. Collections are presented chronologically. Bar graphs (left) show luminal, neuroendocrine (NE), stemness and drug target genes over‐expressed for each collection, denoted by different colours. The panels on the right show blood prostate‐specific antigen (PSA) curves from time of CRPC to last follow‐up, along with treatments administered (RT: radiation therapy; androgen receptor inhibitors: abiraterone, enzalutamide; taxanes: docetaxel, cabazitaxel). In (A, right panel), the testosterone profile is also shown in grey. Vertical dashed lines in the PSA graphs represent the timing of each blood draw. Grey vertical lines at the far right in the trajectory indicate time of death.

Results in samples collected periodically were analysed for a subset of mCRPC patients (Fig. 5). The first patient showed 22 genes over‐expressed at the start of first‐line ARI treatment, with 19 of these genes lost 22 months later while stable on this same line of treatment (Fig. 5A).

Additional cases demonstrate changes in gene patterns in line with progression and changes in treatment. Figure 5B shows a shift in gene profiles between treatment modalities, where genes related to taxane resistance (Fig. 1) come up in samples collected after taxane initiation (*IGFBP2*, *POU3F2*, *KIF2C*), and additional genes of all subtypes appear upon further progression. In another patient (Fig. 5C), a loss of luminal and stemness genes was observed upon switching from taxanes to ARIs, with different NE patterns present at each timepoint.

Finally, a patient post‐ARI with four timepoints on taxanes and progressing based on PSA levels displayed increasing genes from the start of taxane treatment to 16 months representing all cell subtypes, including two genes related to taxane resistance (*IGFBP2*, *KIF2C*) and the drug target *FLT1* (Fig. 5D). At 19 months, upon switching for 1 month to a different taxane, three NE genes remained (*AURKA*, *IGFBP2*, *VEGFA*).

Altogether, changes in gene patterns in the blood of patients were detected over the course of disease, including resistance genes and drug targets, with progression and changes in treatments.

### 3.8 Circulating genes in metastatic cases correlate with clinical characteristics, treatments and outcomes

**Fig. 6:**
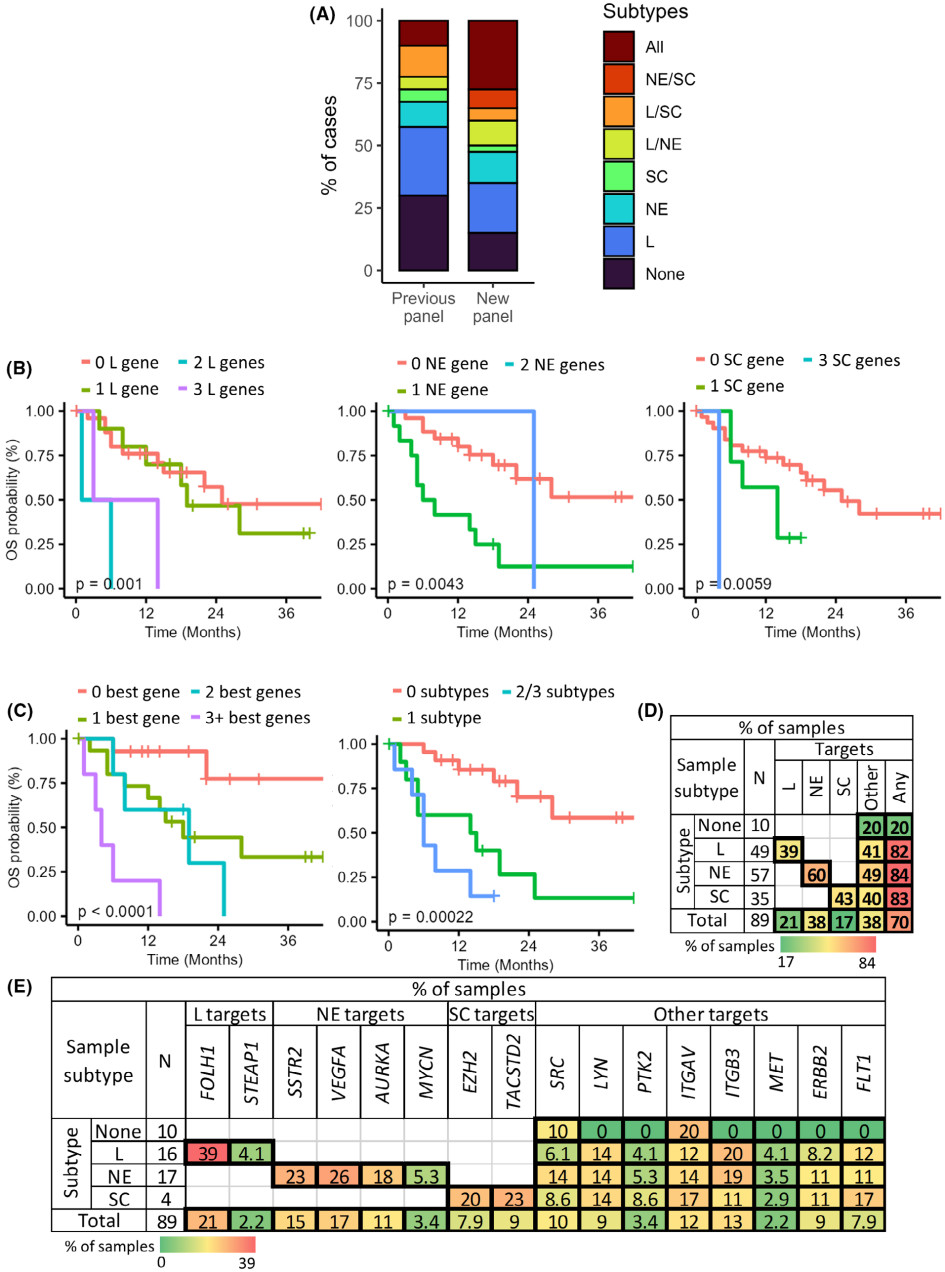
Cell subtype signatures are predictive of outcomes in metastatic patients and drug target genes are commonly over‐expressed throughout all categories of patients. Outcomes in metastatic patients (A–C). (A) Stacked bar graph comparing results using the initial 14 cell‐subtype gene signature vs. all cell‐subtype genes included in the 57‐gene panel. Results are presented as the proportion of cases classified by subtypes or combinations thereof, as indicated by colours on the right. (B, C) Kaplan–Meier curves for overall survival (OS) stratified by (B) the best luminal (L), neuroendocrine (NE) and stemness (SC) signatures (from left to right), (C) the number of genes over‐expressed among these three signatures (left), and the number of cell subtypes represented (right). Log‐rank *P* values are indicated. (D, E) Heatmaps demonstrating the proportion of cases (classified by cell subtype) for which targetable genes are over‐expressed in all patients. The heatmap in (D) summarizes drug targets by cell subtype vs. non‐subtype‐specific (other) or any target, whereas results for individual genes are presented in (E).

While studying individual genes yields valuable results, considering them as signatures of resistance to treatments or cell subtypes better represents the heterogeneity of cancer cells. Classification of patients by cell subtype or combinations thereof revealed that testing additional luminal, NE and stemness genes showed significantly more patients over‐expressing two or more subtypes compared to our initial panel of 14 genes (Fig. 6A; *P* = 0.039).

Survival analyses by cell‐subtype genes showed relevance of PCa‐specific luminal genes (*KLK3*, *AR*, *ARV7*, *GRHL2*, *FOLH1*, *STEAP1*) for overall survival, while the total number of genes and NE or EMT genes were more relevant to progression (Table 3). Patients with genes from all subtypes had shorter time to progression and shorter overall survival. To simplify the cell subtype models, the best 3‐gene signatures for each subtype were considered (luminal: *ARV7*, *KLK3*, *GRHL2*; NE: *AURKA*, *SYP*, *NCAM180*; Stem: *EZH2*, *PROM1*, *KIF2C*). Kaplan–Meier curves (Fig. 6B,C) and Cox analyses (Table 3) showed a stronger effect on both overall survival and disease progression. Looking at broader gene panels, we found by Cox analyses that signatures of ARI resistance (Table 1) and the three best genes related to ARI/taxane resistance (*AURKA*, *IGFBP2*, *KIF2C*) were relevant to both disease progression and overall survival (Table 3).

**Table 3:** Survival analyses by gene signature in metastatic patients. Cox analyses were carried out by gene signature for progression‐free survival (PFS) or overall survival (OS). Significant results are presented in bold (*P* < 0.05). ARI, androgen receptor inhibitor; CI, confidence interval; EMT, epithelial to mesenchymal transition; HR, hazard ratio; NE, neuroendocrine; SC, stem cell.

Category	PFS		OS	
	HR	95% CI	HR	95% CI
Number of genes	**1.07**	**1.00–1.14**	1.00	0.93–1.09
Number of luminal genes	1.13	0.93–1.38	1.17	0.90–1.51
Number of PCa‐specific luminal genes	1.20	0.91–1.58	**1.49**	**1.05–2.09**
Number of general/epithelial luminal genes	1.00	0.68–1.48	0.84	0.53–1.35
Number of NE genes	**1.26**	**1.03–1.54**	1.06	0.84–1.33
Number of stem genes	1.23	0.92–1.64	0.94	0.65–1.35
Number of EMT genes	**1.28**	**1.03–1.59**	1.05	0.81–1.37
Number of stemness and EMT genes	**1.15**	**1.01–1.32**	1.02	0.87–1.20
Any luminal gene	0.83	0.41–1.64	0.92	0.38–2.26
Any PCa‐specific luminal genes	1.14	0.57–2.28	1.28	0.52–3.13
Any general/epithelial luminal genes	0.80	0.40–1.58	0.71	0.29–1.72
Any NE gene	**2.68**	**1.25–5.72**	2.18	0.84–5.69
Any stemness gene	1.61	0.81–3.22	1.24	0.51–2.98
Any EMT gene	**2.48**	**1.16–5.27**	2.19	0.83–5.71
Any stemness or EMT gene	**2.47**	**1.12–5.48**	**3.05**	**1.01–9.15**
Any 2 luminal (PCa), 2 NE and 1 stem gene	2.36	0.69–8.10	**6.42**	**1.77–23.30**
Number of subtypes – 1	**2.82**	**1.07–7.40**	2.81	0.84–9.39
Number of subtypes – 2	**2.60**	**1.03–6.56**	1.44	0.39–5.38
Number of subtypes – 3	**4.97**	**1.20–20.57**	**10.80**	**2.28–51.08**
Any 2 of the 3 best luminal genes	2.48	0.82–7.48	**6.71**	**2.08–21.68**
Any of the 3 best NE genes	**3.54**	**1.46–8.55**	**3.72**	**1.53–9.04**
Any of the 3 best SC genes	1.56	0.66–3.65	**2.77**	**1.00–7.73**
Any 2 of the best genes	**3.40**	**1.39–8.31**	**4.09**	**1.65–10.17**
Number of best genes	**1.43**	**1.11–1.85**	**1.72**	**1.31–2.27**
Number of subtypes – 1	2.24	0.94–5.36	**4.44**	**1.53–12.89**
Number of subtypes – 2/3	**4.10**	**1.44–11.63**	**8.34**	**2.48–28.05**
Number of ARI resistance genes	**1.51**	**1.18–1.94**	**1.43**	**1.07–1.90**
Number of taxane resistance genes	**1.68**	**1.11–2.55**	1.24	0.78–1.95
Number of best resistance genes	**1.78**	**1.21–2.59**	**1.72**	**1.12–2.62**
Any ARI resistance gene	**3.89**	**1.66–9.12**	**3.51**	**1.03–12.00**
Any taxane resistance gene	**2.59**	**1.22–5.48**	2.06	0.85–4.99
Any 2 of the best resistance genes	**2.93**	**1.19–7.24**	**3.76**	**1.47–9.62**

As PSA is the main biomarker for monitoring PCa progression, we performed survival analyses of these signatures in combination with PSA. All signatures when combined with PSA showed improved predictive value compared to PSA alone as shown by Harrel's c‐index (Fig. S7A). However, these signatures combined with PSA do not remain independent predictors of overall survival in Cox multi‐variate analyses (Fig. S7C).

Further analyses by gene signatures (Table 4) showed that luminal and stemness genes were significantly associated with higher PSA, while NE genes were more common in patients with progressive disease. Resistance signatures were associated with higher PSA, more lines of treatment, progressive disease and current taxane treatment, thus being related to more advanced disease overall. Interestingly, the initial treatment also affects gene patterns: NE, EMT and ARI‐resistance genes were less common in cases treated by RP as opposed to radiotherapy or non‐curative treatments, suggesting that these genes were more common in cases who initially presented with more aggressive disease or consequent to RT. Altogether, several PCa‐relevant genes, including genes representative of each PCa cell subtype and genes related to resistance, were over‐expressed in blood of mCRPC patients, with gene patterns associated to clinical features and outcomes.

**Table 4:** Comparison of circulating gene signatures associated with clinical features and treatments in metastatic patients. Mixed‐effects logistic regression for gene signatures in blood vs. clinical parameters and treatments in metastatic patients. Significant results are presented in bold. ARI, androgen receptor inhibitor; CI, confidence interval; EMT, epithelial to mesenchymal transition; HR, hazard ratio; PSA, prostate‐specific antigen; RT, radiation therapy.

HR (95% CI)	Luminal	Neuroendocrine	Stemness	Stem/EMT	ARI resistance	Taxane resistance
Age^a^	0.66 (0.22–2.01)	1.28 (0.51–3.24)	1.82 (0.48–6.81)	1.77 (0.58–5.36)	0.91 (0.37–2.19)	1.86 (0.59–5.90)
PSA^a^	**3.11 (1.12–8.64)**	1.85 (0.83–4.12)	**4.00 (1.04–15.28)**	**4.54 (1.48–13.92)**	**4.00 (1.98–8.09)**	**3.93 (1.25–12.35)**
Initial therapy						
Surgery	0.43 (0.11–1.75)	**0.18 (0.05–0.70)**	0.11 (0.01–1.13)	**0.13 (0.03–0.64)**	**0.18 (0.05–0.66)**	0.14 (0.02–1.12)
Curative RT	1.13 (0.33–3.80)	1.15 (0.42–3.14)	1.22 (0.31–4.79)	1.21 (0.36–4.07)	0.63 (0.24–1.65)	0.85 (0.25–2.95)
Non‐curative	1.34 (0.44–4.10)	1.54 (0.60–3.91)	2.01 (0.54–7.51)	1.96 (0.65–5.92)	**2.50 (1.10–5.70)**	2.00 (0.68–5.88)
Any pelvic RT	1.31 (0.42–4.10)	1.30 (0.50–3.36)	1.36 (0.38–4.92)	1.55 (0.51–4.71)	0.62 (0.26–1.45)	0.72 (0.24–2.20)
Past mCRPC therapy						
ARI	0.41 (0.12–1.43)	1.06 (0.40–2.81)	0.74 (0.19–2.83)	0.95 (0.30–3.01)	1.67 (0.70–3.99)	2.55 (0.88–7.34)
Taxane	0.80 (0.29–2.22)	1.09 (0.50–2.36)	1.06 (0.37–3.06)	0.70 (0.31–1.60)	1.62 (0.78–3.39)	1.10 (0.38–3.19)
Current therapy						
ARI	1.56 (0.56–4.30)	0.62 (0.26–1.46)	0.82 (0.25–2.68)	0.89 (0.39–2.07)	0.56 (0.26–1.21)	0.66 (0.23–1.95)
Taxane	1.20 (0.43–3.40)	1.74 (0.76–4.00)	2.51 (0.61–10.41)	1.65 (0.65–4.20)	**2.72 (1.31–5.66)**	2.63 (0.91–7.60)
Line of therapy	0.89 (0.50–1.61)	1.24 (0.77–1.99)	1.16 (0.63–2.16)	0.94 (0.58–1.53)	**1.57 (1.04–2.37)**	1.54 (0.88–2.69)
Progressive disease	2.01 (0.80–5.05)	**2.51 (1.18–5.32)**	1.47 (0.53–4.02)	**2.44 (1.13–5.26)**	**4.31 (2.19–8.49)**	**3.81 (1.36–10.71)**

a Patients were stratified below and above the median.

### 3.9 Validation

**Fig. 7:**
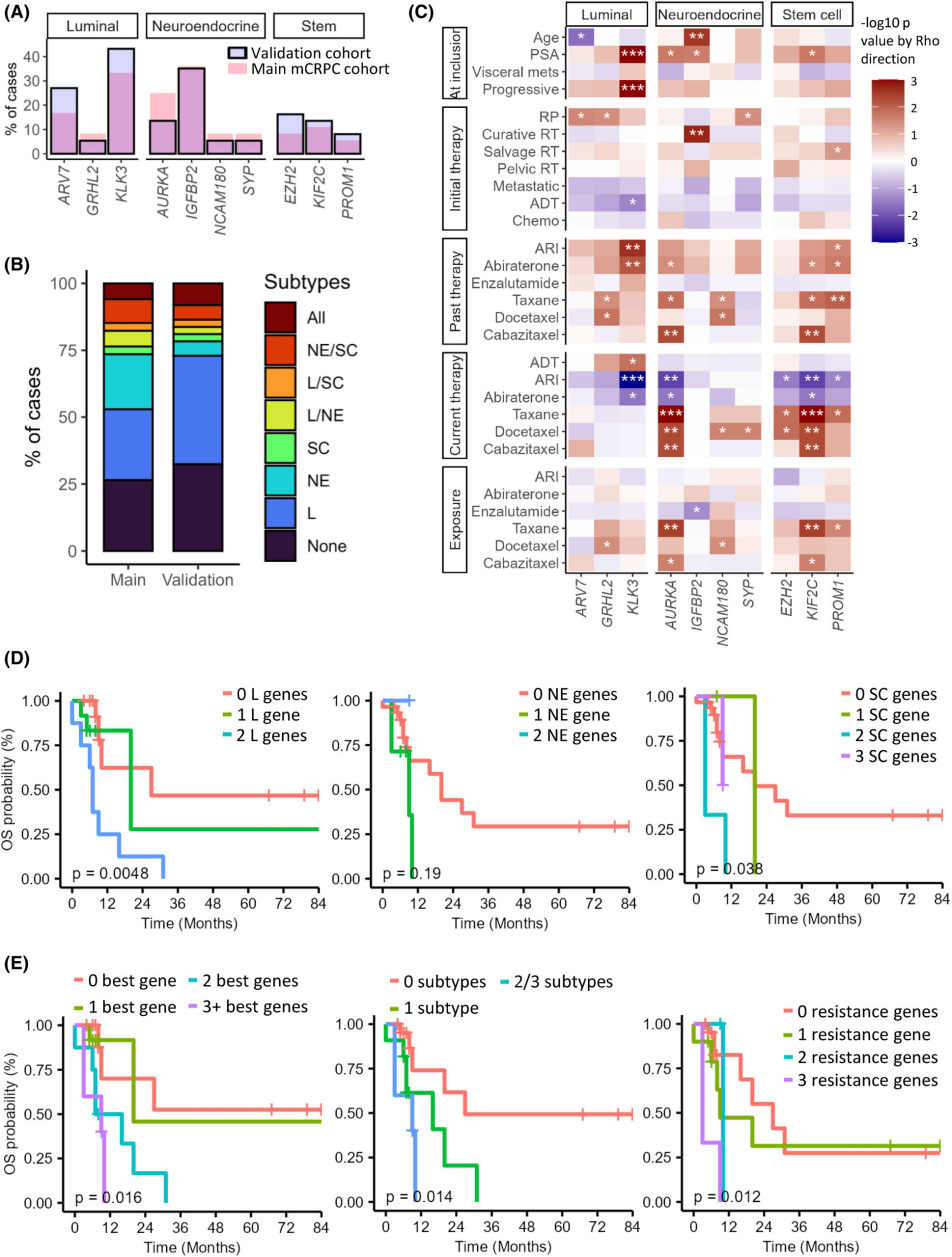
Cell subtype and resistance signatures are predictive of outcomes in a validation cohort of mCRPC patients. (A) Bar graph representing the proportion of cases over‐expressing each gene among metastatic castration resistant prostate cancer (mCRPC) cases of the main cohort (pink bars) and the validation cohort (blue bars). No significant differences were found by χ^2^ test. (B) Stacked bar graph comparing the proportion of cases classified by subtypes or combinations thereof, using only the three best genes per subtype, in the main cohort and the validation cohort. (C) Heatmap of χ^2^ results for associations between over‐expression of genes in the mCRPC validation cohort and clinical characteristics or treatments. Red and blue represent positive and negative correlations, respectively. Significant associations are indicated: * for *P* < 0.05; ** for *P* < 0.01; *** for *P* < 0.001. (D, E) Kaplan–Meier curves for overall survival (OS) in the mCRPC validation cohort, stratified by (D, from left to right) the best luminal (L), neuroendocrine (NE) and stemness (SC) signatures, (E, from left to right) the number of genes over‐expressed among these three signatures, the number of cell subtypes represented or the number of resistance related genes. Log‐rank *P* values are indicated. ADT, androgen deprivation therapy; ARI, androgen receptor inhibitor; PSA, prostate‐specific antigen; RP, radical prostatectomy; RT, radiation therapy.

The three best genes by cell subtype, as well as the best genes related to ARI/taxane resistance, were tested in a validation cohort of 32 mCRPC patients. The proportion of mCRPC cases over‐expressing each of these genes was not different between cohorts (Fig. 7A). Categorizing patients using only the nine best genes by subtype, we see that the validation cohort consists of more luminal cases and fewer NE cases compared to the original mCRPC cohort (Fig. 7B).

Results of individual genes vs. patients' clinical features and treatments were in line with the original cohort (Fig. 7C), showing *AURKA* and *KIF2C* in cases with taxane exposure. Interestingly, *KLK3* was the most significant gene in the validation cohort, showing positive correlation with higher PSA and progressive disease at inclusion, in agreement with our pilot study [7].

In survival analyses by gene, *AURKA* remained associated with progression‐free survival and overall survival (Table S3). While *GRHL2* and *IGFBP2* were no longer significantly associated with outcomes in the validation cohort, we found *ARV7* to be associated with overall survival, while *KIF2C* and *KLK3* with both progression‐free survival and overall survival (Table S3).

Most importantly, survival analyses by cell subtypes and resistance genes yielded results in line with the main cohort, although luminal genes alone were more clinically relevant in this cohort as opposed to NE genes (Table 5, Fig. 7D,E). Survival analyses of these signatures in combination with PSA showed that they increased the predictive power of PSA for overall survival (Fig. S7B,C).

**Table 5:** Survival analyses by genes and signature in mCRPC patients of validation cohort. Cox analyses were carried out by gene signature for progression‐free survival (PFS) or overall survival (OS). Significant results are presented in bold (*P* < 0.05). CI, confidence interval; HR, hazard ratio; NE, neuroendocrine; SC, stem cell.

Category	PFS		OS	
	HR	95% CI	HR	95% CI
Any 2 of the 3 best luminal genes	**3.00**	**1.27–7.12**	**4.33**	**1.62–11.59**
Any of the 3 best NE genes	2.34	0.96–5.66	2.22	0.64–7.66
Any of the 3 best SC genes	**2.65**	**1.06–6.61**	2.47	0.81–7.52
Any 2 of the best genes	**4.20**	**1.82–9.67**	**4.78**	**1.63–14.02**
Number of best genes	**2.23**	**1.53–3.27**	**1.46**	**1.11–1.92**
Number of subtypes – 1	**3.36**	**1.38–8.22**	**3.45**	**1.08–11.06**
Number of subtypes – 2/3	**4.56**	**1.47–14.12**	**6.57**	**1.54–28.0**
Number of best resistance genes	**1.56**	**1.07–2.27**	**1.91**	**1.14–3.20**
Any 2 of the best resistance genes	**2.77**	**1.00–6.68**	**3.60**	**1.04–12.48**

Overall, results in this validation cohort further support the clinical relevance of tracing genes representative of cell subtypes and mechanisms of resistance in the blood of patients with mCRPC.

### 3.10 Genes encoding drug targets are commonly over‐expressed

As 23/57 genes were targets of drugs tested in clinical trials for PCa, 11 of which were also cell‐subtype‐specific, we questioned whether they were commonly over‐expressed (Fig. 6D). Drug target genes were detected in 70% of samples overall; 39–60% of samples over‐expressed drug target genes specific to a subtype and 38% showed targets not specific to a subtype, including 20% of cases with no cell‐subtype genes represented. The most common targetable genes by subtype were *FOLH1*, *VEGFA* and *TACSTD2* (Fig. 6E). *ITGAV*, *ITGB3* and *FLT1* were also notable in the category of other drugs. Overall, genes encoding drug targets are commonly over‐expressed in liquid biopsies of PCa patients and may be relevant for choosing optimal personalized treatments.

## 4 Discussion

This investigation on the over‐expression of a panel of 57 circulating genes representing PCa cell subtypes, treatment resistance and alternative therapeutic targets demonstrates clinical relevance, thereby opening a path to better patient stratification from diagnosis to late stages of disease. These findings agree with our earlier study limited to 14 cell subtype genes in 40 other mCRPC cases and 40 healthy controls [7].

Liquid biopsies have grown increasingly popular for assessing molecular changes in tumours during the course of disease. Commonly studied analytes include CTCs [19, 20, 21], EVs [22] and cfDNA [23], each with different usefulness, opportunities and limitations mainly pertaining to their isolation. We chose to study whole‐blood RNA to encompass all blood components and overcome these limitations, being aware of other challenges due to various blood components. This approach remains purposefully agnostic to the origin of the transcripts, relying heavily on the choice of genes and methodology. Studies of cancer‐specific transcripts in whole blood as non‐invasive markers of disease by RT‐PCR date back to the 1990s, including *KLK3* in PCa [24]. Gene expression microarray and RNA‐sequencing focusing on a global view of changes in leucocytes have shown interesting results in various cancers. Searching for cancer‐specific genes by these approaches has yielded minimal results due to low expression, as opposed to qPCR which provides a wider dynamic range of quantitative detection. We opted for TaqMan‐based RT‐qPCR assays, providing higher sensitivity and specificity than SYBR Green‐based assays. Extensive technical and biological validation were carried out and showed consistently quantifiable and reproducible results with minimal expression in controls.

Our strategy for building the gene panel was crucial in order to specify genes relevant to the disease and not influenced by normal inter‐individual differences, fluctuations in WBC populations or changes with aging or lifestyle. The panel was curated to mirror various aspects of advanced disease, including genes encoding drug targets. Despite their relation to PARP inhibitor sensitivity, DNA repair genes such as *BRCA1*/*2*, *ATM* and *CHEK2* were not included, as their relevance is mainly due to mutations therein which may be best studied in cfDNA. Due to our exclusion criteria, certain genes of interest were not considered, such as the stemness gene *ITGB1*, expressed in WBCs and platelets; the prostate‐specific marker *ACP3* (prostatic acid phosphatase), involved in PCa bone metastasis but expressed in eosinophils; and the EMT marker *VIM*, highly expressed in WBCs, which is more relevant in CTCs than in whole‐blood RNA [25, 26, 27]. We also considered splice variants specific to cancer and less expressed in blood (e.g., *NCAM180*, *CD44E* and *FYN‐B*), resulting in higher specificity and lower background expression. Overall, this thorough selection criteria proved to be most valuable; our results did not correlate to WBCs or age, while several genes and signatures correlated with clinical and pathological characteristics and treatments. Although we limited the panel to prostate‐ and cancer‐specific genes, it may be expanded to reflect systemic immune signatures related to treatment response or coupled to longitudinal testing of cfDNA, EVs or CTCs.

Circulating cancer‐specific transcripts likely originate from CTCs and EVs released from cancer cells. For example, over‐expression of *AURKA* and *KIF2C* in blood of metastatic patients exclusively may be due to their over‐expression specifically in advanced disease. *KLK3* and *FOLH1* are expressed in tumours even in early disease and may be representative of disease burden. Interestingly, while circulating *FOLH1* is detected even at diagnosis, *KLK3* is exclusively detected in mCRPC cases. This may be due to the lower levels of CTCs/cancer‐EVs released in blood at these early stages. Differences in assay sensitivities cannot be excluded. Sensitivity appears to be an issue for genes such as *EGFR*, which was not detected in any blood samples but is a common marker of CTCs in PCa [28, 29]. Further investigation using more sensitive assays, such as digital PCR or targeted sequencing, deserves consideration to address this issue.

Our results are in line with the concept of luminal features reflecting tumour burden, with circulating luminal genes being significantly more common in metastatic patients. While NE and stemness features are known to increase with stage and upon treatments, the predominating cells in most tumours remain luminal‐like, as observed in this investigation and as we recently reported [2]. This is further evidenced by blood PSA, a marker of inherently luminal‐like AR+ PCa cells, reflecting disease progression even at late stages for most patients. The finding that ARI resistance genes are most common in mCRPC cases is in line with the ADT sensitivity at the HSPC stage.

An important concern is whether circulating genes may help to stratify PCa patients at diagnosis and upon recurrence. While this panel was mainly curated to study advanced disease, cell‐subtype genes were relevant for patient stratification at diagnosis. Patients with higher PSA at diagnosis over‐expressed more genes and only intermediate‐ and high‐risk patients over‐expressed NE genes. The main findings here showed that cases with IDC, which is associated with poor outcomes [30], over‐expressed more circulating genes, especially NE and stemness genes. This shows potential as a non‐invasive predictor of IDC, alongside recent findings in cfDNA and multi‐parametric MRI, which may be relevant for patients undergoing non‐surgical interventions [31, 32]. Further follow‐up is necessary to determine the relevance of these genes for outcomes after RP. While the patients tested at diagnosis underwent RP, this approach can also be applied to presumably low‐risk cases offered active surveillance, focal therapy or curative radiotherapy, as well as for assessing minimal residual disease after surgery.

Testing specific circulating genes in metastatic patients is promising to offer tailored therapies. We found that *AURKA* was highly predictive of progressive disease at inclusion and was the best single gene to predict outcomes in Cox analyses. It came up mainly in cases who progressed on ARIs and taxanes and who were on taxanes at the time of blood draw. AURKA is a potential target for metastatic NEPC where it is amplified, being involved in genomic instability via its role in mitosis. A phase II trial of alisertib showed promising results, where 4/60 unselected patients had exceptional response and were confirmed to have AURKA/NMYC overactivity [33]. Here, we found circulating *MYCN* over‐expression in very few cases, irrespectively of *AURKA* over‐expression. An alternate cfDNA‐based assay for *MYCN* amplification may also be considered. Evaluating disruptions in AURKA/NMYC in this manner could be useful in the context of clinical trials to either stratify patients without the need for tumour biopsies or for longitudinal monitoring to assess response to these treatments.

Another remarkable NE gene in mCRPC cases was *SSTR2*, over‐expressed exclusively in patients who received pelvic radiotherapy. Radiation is known to induce NE differentiation in PCa [34]. Increased SSTR2 has been reported upon radiation in other cancers, making SSTRs good targets for theranostics of various NE tumours and potentially applicable to NE‐enriched PCa [35, 36, 37]. Considering the introduction of PSMA theranostics for advanced PCa, further studies are needed to assess *FOLH1* vs. *SSTR2* over‐expression in blood of patients as indicators of intra‐tumoural heterogeneity and predictors of response to their respective radionuclide therapies [38]. In this study, we found *SSTR2* and *FOLH1* exclusively in 11 and 16 patients, whereas two cases over‐expressed both.

*KIF2C* was another central gene over‐expressed in the blood of patients treated with taxanes at diagnosis or who were on taxanes at enrolment. We considered *KIF2C* as a stemness‐related gene based on studies in breast, kidney and bladder cancer [39, 40, 41, 42]. It is also a potential therapeutic target over‐expressed in both castration‐resistant and docetaxel‐resistant PCa [43]. *KIF2C* over‐expression was reported upon paclitaxel resistance, including in triple‐negative breast cancer where its inhibition sensitized cells to paclitaxel [44]. Conversely, it may be a target of cabazitaxel according to *in vitro* and *in vivo* findings in PCa [45, 46]. Our study on a limited series of cases did not allow us to explore differences between docetaxel and cabazitaxel responsiveness.

While the full panel represents the heterogeneity found among PCa cells, a limited set of nine genes, chosen as the most relevant to survival for each subtype, was highly predictive of time to progression and overall survival. These included genes significantly more common in metastatic cases. These genes are the most relevant in terms of outcomes, while the complete panel is more suitable for classification of patients by cell subtype and guiding treatment decisions.

Genes related to ARI and taxane resistance also predicted progression and survival, suggesting that investigating specific resistance mechanisms may be helpful to better stratify and treat patients, especially as some of these genes are targetable. Mixed‐effects models showed the relevance of these gene sets, where all were over‐represented in cases with higher PSA and progressive disease. ARI resistance genes came up mostly in heavily treated patients currently on taxanes, in line with their previous progression on ARIs. Interestingly, NE, EMT/stem and ARI resistance signatures were under‐represented in patients initially treated by surgery as opposed to radiation or non‐curative therapies.

Our results were validated in a cohort of mCRPC patients for the best genes representative of cell subtypes and resistance. The validation cohort tended to have patients of lower grade at diagnosis, as well as a lower proportion of cases currently on or exposed to taxanes, with overall more lines of treatment at time of inclusion. These differences are reflected in their over‐expressed genes, where the validation cohort shows more luminal subtypes vs. NE in the main cohort. This may explain why PSA seems to be more predictive of outcomes in the validation cohort. Overall, these results further corroborated our findings, especially for the number of subtypes represented in the blood.

Testing our gene set in patients with repeated blood collections revealed different evolving patterns modified by treatments and progression. In patients post‐RP, some prostate‐specific genes remain over‐expressed despite no signs of recurrence and may be indicative of minimal residual disease. Gene patterns and subtypes also shift with progressive disease, RT and ADT. In metastatic cases, while some genes remain over‐expressed throughout multiple collections from a case, others fluctuate, disappear with changes in treatments or appear with progression, including genes related to resistance to the relevant treatments. The relevance of these observations is meaningful despite limitations due to the low number of patients and the timing of blood draws, particularly in metastatic cases. Ideally, blood should be collected before treatment initiation, at the time of resistance and progression and with a change of therapy. Such well‐defined cohorts are necessary to refine signatures. Furthermore, the fact that genes encoding various targets (such as *FOLH1*, *AURKA*, *SSTR2* and *TACSTD2*) become apparent in mCRPC patients and show clinical relevance alone or as part of signatures supports the suitability of this approach to initiate clinical trials establishing which patients would best benefit from the corresponding drugs, paving the path to therapies tailored to patients' characteristics.

## 5 Conclusion

This study in two cohorts substantiates the relevance of circulating PCa‐relevant genes in whole‐blood RNA of patients for following phenotypic and functional changes non‐invasively throughout the disease trajectory. Our findings revealed associations of cell subtype‐specific genes in patients' blood at diagnosis with worse pathological features, suggesting they may benefit from more aggressive treatment. At the metastatic stage, circulating genes relevant to cell subtypes, resistance to current therapeutic modalities or encoding alternate drug targets correlated with clinical parameters and treatments and were related to outcomes. Altogether, a better stratification of patients will allow for new clinical trials bringing us closer to personalized therapies for a heterogeneous, unpredictable and lethal disease.

## Conflict of interest

The authors declare no conflict of interest.

## Author contributions

SC and SD designed the project and were involved in all steps. RS‐S, AR‐B, WK, RR, FB, MD and AA provided clinical information and discussed clinical relevance of results. LH periodically updated patients' clinical information. SD, EJ, AS, QV and LH processed and banked blood samples. SD, EJ and AS optimized assays and performed RT‐qPCR experiments. SD performed bioinformatic and statistical analyses. SD, SC and AA wrote and edit the manuscript and also reviewed by all co‐authors.

### Peer review

The peer review history for this article is available at https://www.webofscience.com/api/gateway/wos/peer‐review/10.1002/1878‐0261.13805.

## Supporting information


**Fig. S1.** Gene expression is in line with results in cell lines and shows consistent results throughout batches of RNA extraction and cDNA synthesis.

**Fig. S2.** Longitudinal testing of selected genes in blood of healthy controls.

**Fig. S3.** Reproducibility of gene expression in patients' blood RNA.

**Fig. S4.** Comparison of patients' blood cell counts vs. gene expression.

**Fig. S5.** Gene expression patterns in PCa patients at different stages of disease.

**Fig. S6.** Clinical relevance of circulating genes over‐expressed in patients upon recurrence.

**Fig. S7.** Circulating gene signatures increase the predictive value of PSA in metastatic patients.

**Table S1.** Clinical and pathological characteristics of patients included in the study.

**Table S2.** Assay specifications and efficiencies for selected genes.

**Table S3.** Cox analyses by gene for overall survival and progression‐free survival.

## Data Availability

The data that support the findings of this study are available on request from the corresponding author. The data are not publicly available due to privacy or ethical restrictions.
