# Supplementary material for: Clinical significance of stratifying prostate cancer patients through specific circulating genes
Source: Mol Oncol. 2025 Jan 22;19(5):1310–31. doi: 10.1002/1878-0261.13805 (PMC12077267; doi:10.1002/1878-0261.13805)
Supplement: Supplementary file 1 — Fig. S1. Gene expression is in line with results in cell lines and shows consistent results throughout batches of RNA extraction and cDNA synthesis. [file MOL2-19-1310-s006.pdf]

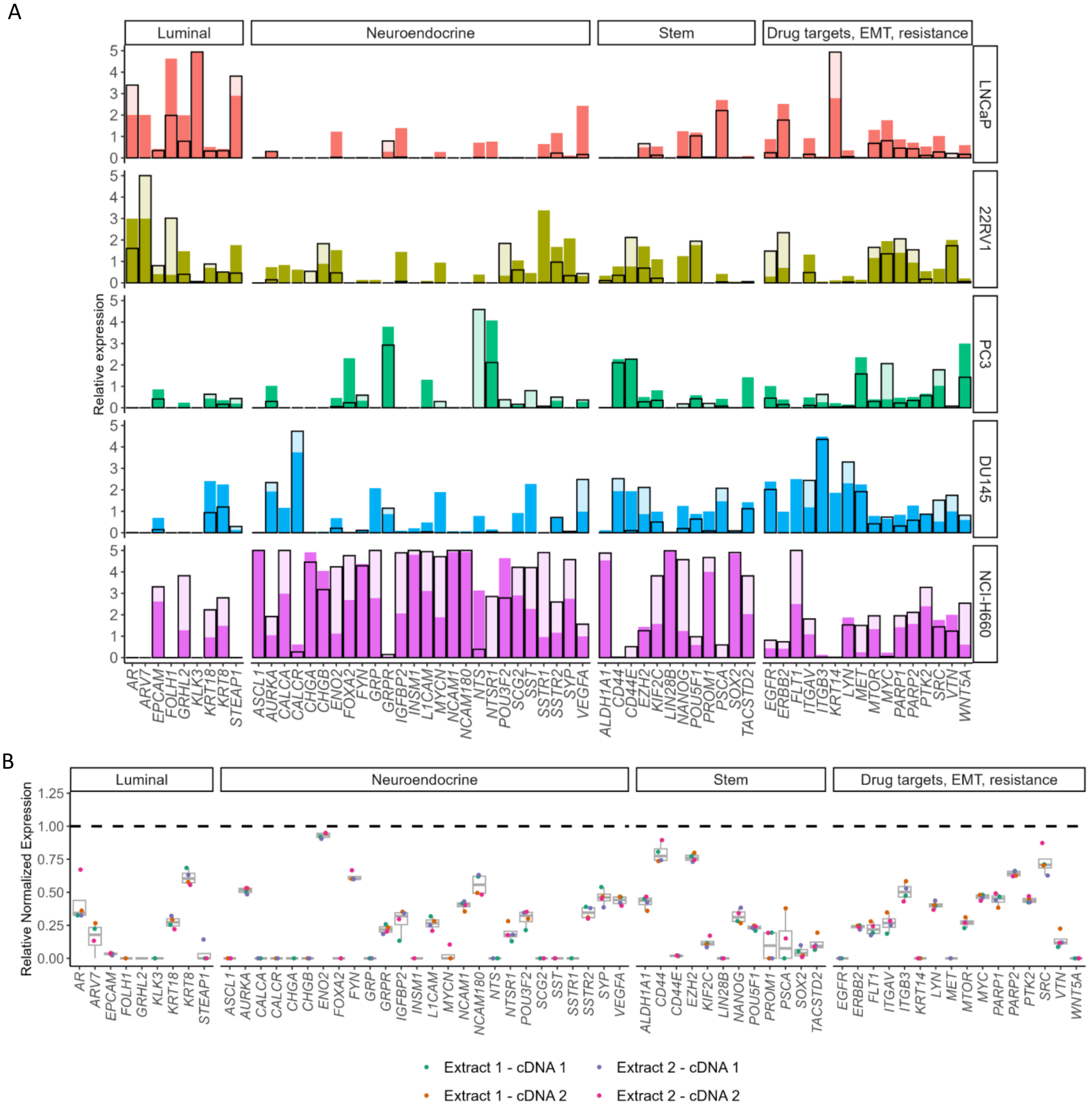

Figure S1: Gene expression is in line with results in cell lines and shows consistent results throughout batches of RNA extraction and cDNA synthesis.

(**A**) Genes were tested by RT-qPCR in RNA of five PCa cell lines and compared to expected expression based on RNA-seq results from the CCLE dataset. Results are presented as relative expression among the five cell lines, based on 2^-dCt for qPCR results and TPM for RNA-seq results. Contoured bars represent qPCR data and filled bars represent RNA-seq data. Genes marker with an x were tested as specific splice variants and are not expected to correlate with RNA-seq data. (**B**) Each gene was tested in a single control blood collection from which two batches of RNA extraction and two batches of cDNA synthesis were performed. Extractions and cDNA synthesis were performed on separate days and using separate kits. Expression is presented as the fold change from overexpression threshold (dashed line).
