# Supplementary material for: Clinical significance of stratifying prostate cancer patients through specific circulating genes
Source: Mol Oncol. 2025 Jan 22;19(5):1310–31. doi: 10.1002/1878-0261.13805 (PMC12077267; doi:10.1002/1878-0261.13805)
Supplement: Supplementary file 2 — Fig. S2. Longitudinal testing of selected genes in blood of healthy controls. [file MOL2-19-1310-s008.pdf]

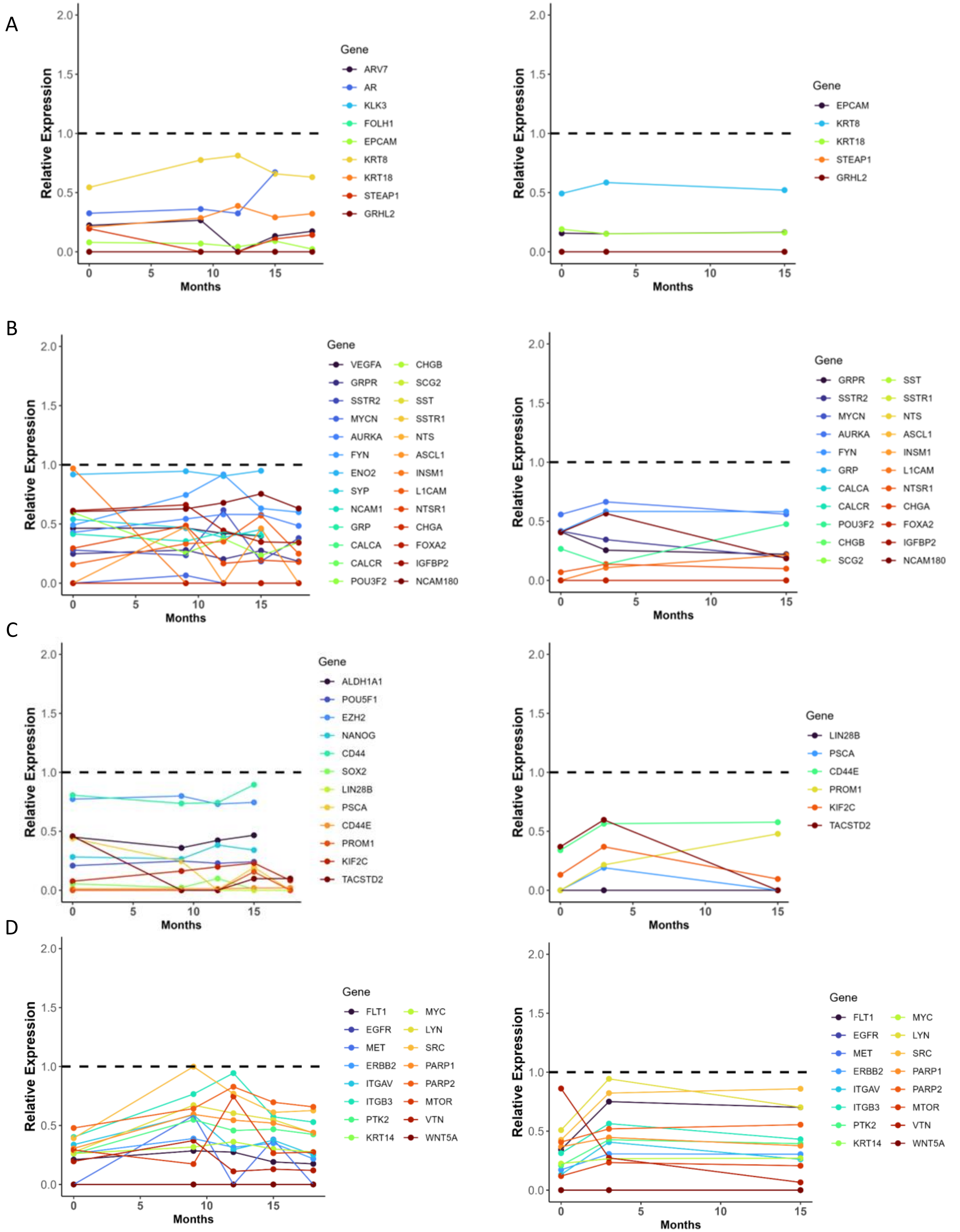

Figure S2: Longitudinal testing of selected genes in blood of healthy controls.
Circulating genes were tested in longitudinally collected blood samples from two controls (left and right panels). Results are presented as fold change from the overexpression threshold for (A) luminal, (B) neuroendocrine, (C) stemness, and (D) drug targets, EMT, and resistance genes.
