# Supplementary material for: Clinical significance of stratifying prostate cancer patients through specific circulating genes
Source: Mol Oncol. 2025 Jan 22;19(5):1310–31. doi: 10.1002/1878-0261.13805 (PMC12077267; doi:10.1002/1878-0261.13805)
Supplement: Supplementary file 4 — Fig. S4. Comparison of patients' blood cell counts vs. gene expression. [file MOL2-19-1310-s010.pdf]

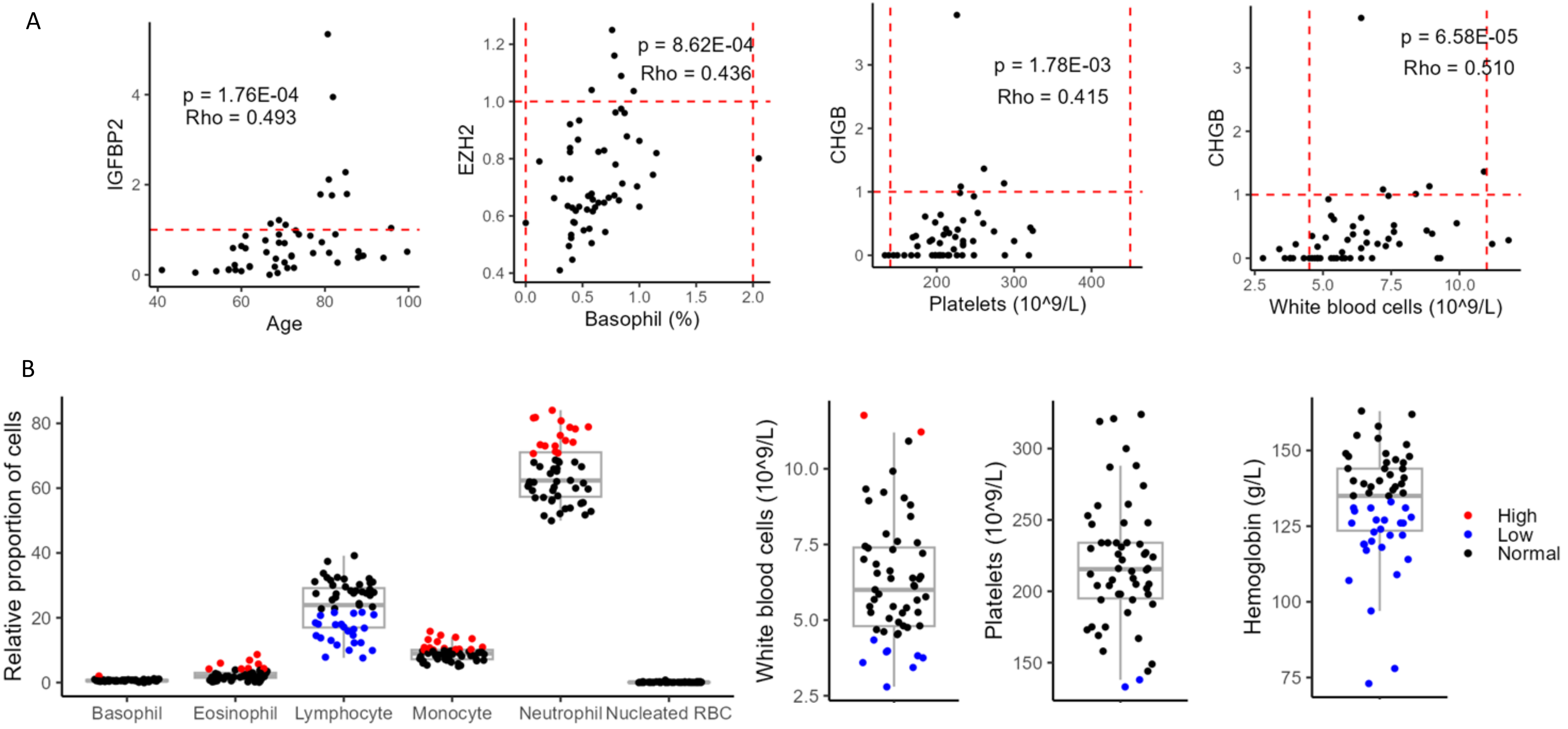

Figure S4: Comparison of patients' blood cell counts vs. gene expression.
(**A**) Scatter plots for genes vs. parameters that were significantly positively correlated (see Figure 2B, indicated by *). The horizontal red dashed line represents the overexpression threshold; vertical red dashed lines represent normal limits for blood components. (**B**, left to right) Relative proportions of white blood cell subpopulations, total WBCs, platelets, and hemoglobin in patients' blood at the time of blood draw. Black dots represents normal values, while red and blue represent higher or lower than the normal range, respectively.
