# Supplementary material for: Clinical significance of stratifying prostate cancer patients through specific circulating genes
Source: Mol Oncol. 2025 Jan 22;19(5):1310–31. doi: 10.1002/1878-0261.13805 (PMC12077267; doi:10.1002/1878-0261.13805)
Supplement: Supplementary file 5 — Fig. S5. Gene expression patterns in PCa patients at different stages of disease. [file MOL2-19-1310-s004.pdf]

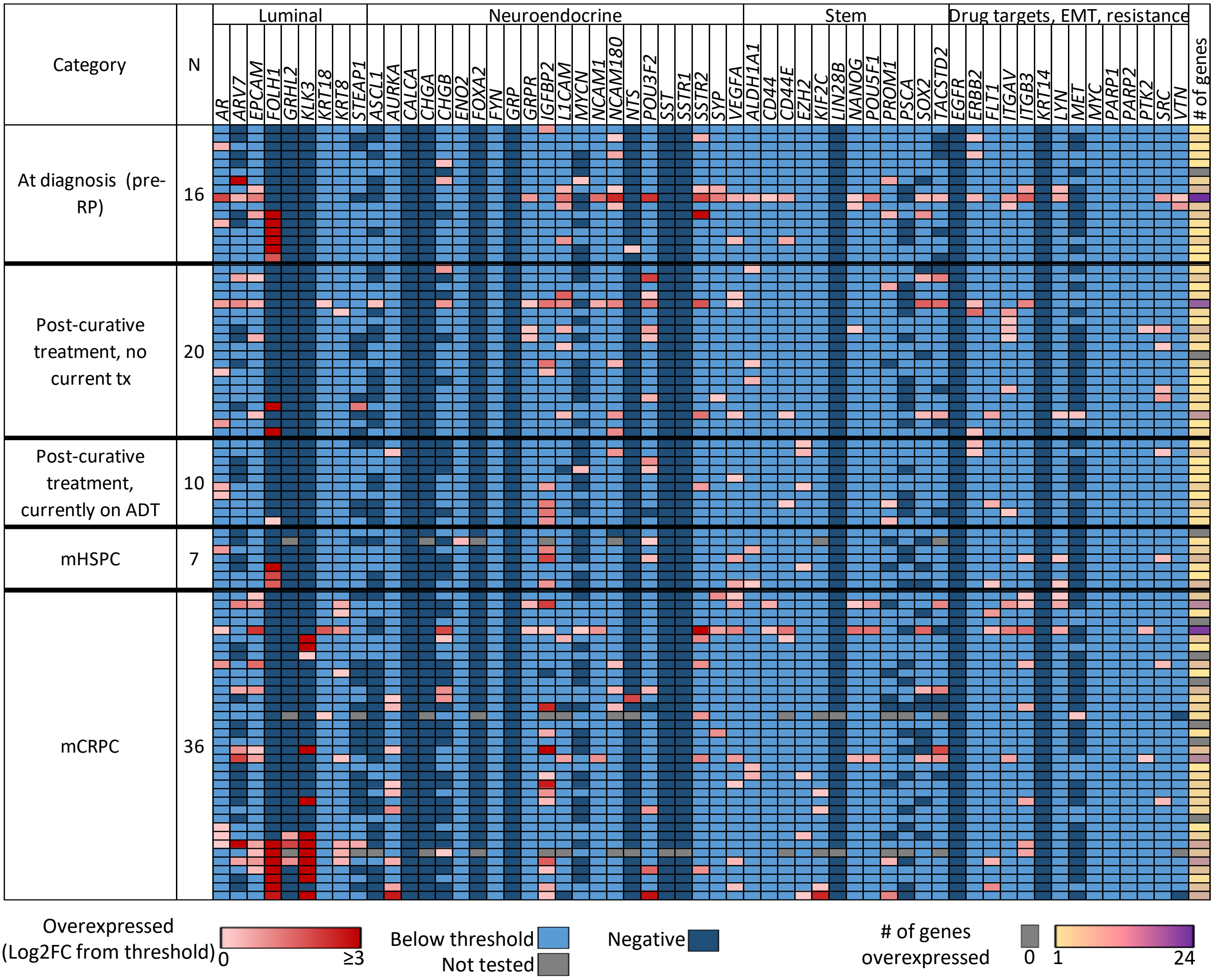

Figure S5: Gene expression patterns in PCa patients at different stages of disease.
Heatmap presenting gene expression results in all categories of patients. Gene overexpression is presented as a log2 fold change from the threshold (gradient of pink to red). For genes with no expression in controls (e.g. *KLK3* and *FOLH1*), the gradient represents the fold change from the lowest positive signal detected in patients. Light blue represents expression below overexpression threshold for each gene, and dark blue represents no signal. Grey represents genes not tested in cases with insufficient RNA. The number of overexpressed genes per patient is presented on the right (gradient of yellow to purple). Only 57/63 genes showing consistent results are presented, as described in Figure S3.
