# Supplementary material for: Clinical significance of stratifying prostate cancer patients through specific circulating genes
Source: Mol Oncol. 2025 Jan 22;19(5):1310–31. doi: 10.1002/1878-0261.13805 (PMC12077267; doi:10.1002/1878-0261.13805)
Supplement: Supplementary file 6 — Fig. S6. Clinical relevance of circulating genes over‐expressed in patients upon recurrence. [file MOL2-19-1310-s002.pdf]

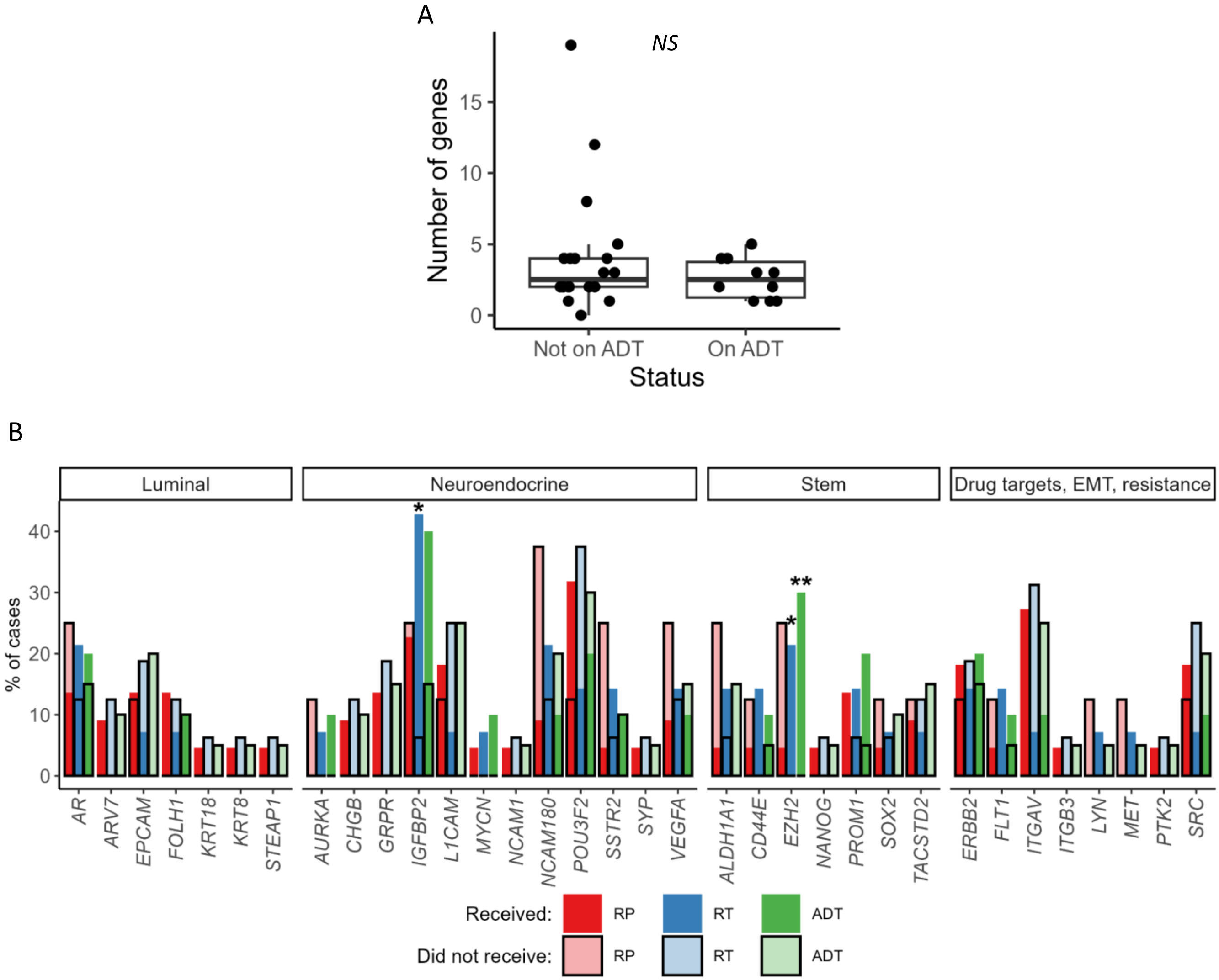

Figure S6: Clinical relevance of circulating genes overexpressed in patients upon recurrence. (A) Box plot representing the total number of genes overexpressed in relation to current ADT treatment in non-metastatic patients with recurrence post-curative therapies (NS: not significant). (B) Bar graph representing the proportion of cases overexpressing each gene vs. whether they have undergone specific treatments (RP, RT, and ADT; in solid dark bars), or not (light bars with black border). Significant Chi2 p-values are denoted by * for p<0.05; ** for p<0.01.
