# Supplementary material for: Clinical significance of stratifying prostate cancer patients through specific circulating genes
Source: Mol Oncol. 2025 Jan 22;19(5):1310–31. doi: 10.1002/1878-0261.13805 (PMC12077267; doi:10.1002/1878-0261.13805)
Supplement: Supplementary file 7 — Fig. S7. Circulating gene signatures increase the predictive value of PSA in metastatic patients. [file MOL2-19-1310-s009.pdf]

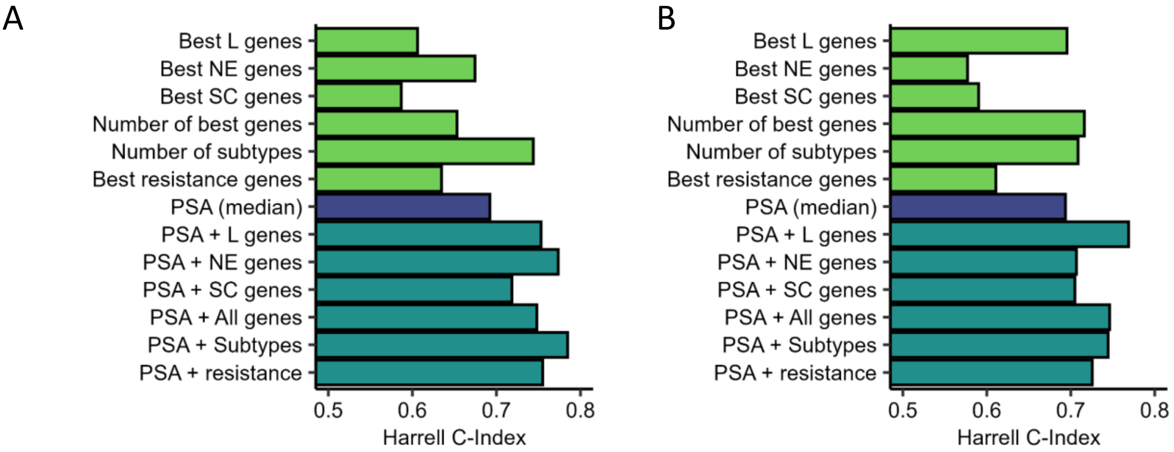

C

Variable	Main cohort				Validation cohort			
	PSA		Other marker		PSA		Other marker	
	HR	95% CI	HR	95% CI	HR	95% CI	HR	95% CI
PSA	**4.45**	**1.80-11.00**	--	--	**9.36**	**2.01-43.55**	--	--
Any 2 of the 3 best luminal genes	**4.06**	**1.47-11.21**	**3.50**	**1.03-11.92**	**8.15**	**1.65-40.26**	**3.29**	**1.18-9.17**
Any of the 3 best NE genes	**3.56**	**1.21-10.52**	2.05	0.76-5.56	**9.19**	**1.92-43.96**	1.09	0.31-3.83
Any of the 3 best SC genes	**4.51**	**1.61-12.62**	1.38	0.46-4.12	**9.33**	**1.87-46.53**	1.01	0.31-3.23
Any 2 of the best genes	**3.66**	**1.28-10.50**	2.37	0.87-6.51	**5.87**	**1.08-31.96**	2.34	0.69-7.95
Number of subtypes – 1	2.52	0.78-8.13	3.04	0.94-9.81	**6.39**	**1.20-34.08**	1.92	0.54-6.80
Number of subtypes - 2/3			**4.28**	**1.01-18.21**			2.52	0.52-12.16
Any 2 of the best resistance genes	**4.11**	**1.50-11.23**	2.45	0.90-6.63	**8.39**	**1.72-40.82**	1.64	0.46-5.85

Figure S7: Circulating gene signatures increase the predictive value of PSA in metastatic patients.

(**A, B**) Harrel C-index for gene signatures (light green), PSA (dark blue), and the combination of PSA + each gene signature (dark green). Results are shown for the main cohort (**A**) and the validation cohort (**B**).

(**C**) Multivariate Cox analyses were carried out for PSA with each gene signature for overall survival (OS) for both the main cohort and the validation cohort. Significant results are presented in bold ($p < 0.05$). Abbreviations: CI: confidence interval; HR: hazard ratio; NE: neuroendocrine; PSA: prostate specific antigen; SC: stem cell.
