# Supplementary material for: Clinical significance of stratifying prostate cancer patients through specific circulating genes
Source: Mol Oncol. 2025 Jan 22;19(5):1310–31. doi: 10.1002/1878-0261.13805 (PMC12077267; doi:10.1002/1878-0261.13805)
Supplement: Supplementary file 3 — Fig. S3. Reproducibility of gene expression in patients' blood RNA. [file MOL2-19-1310-s001.pdf]

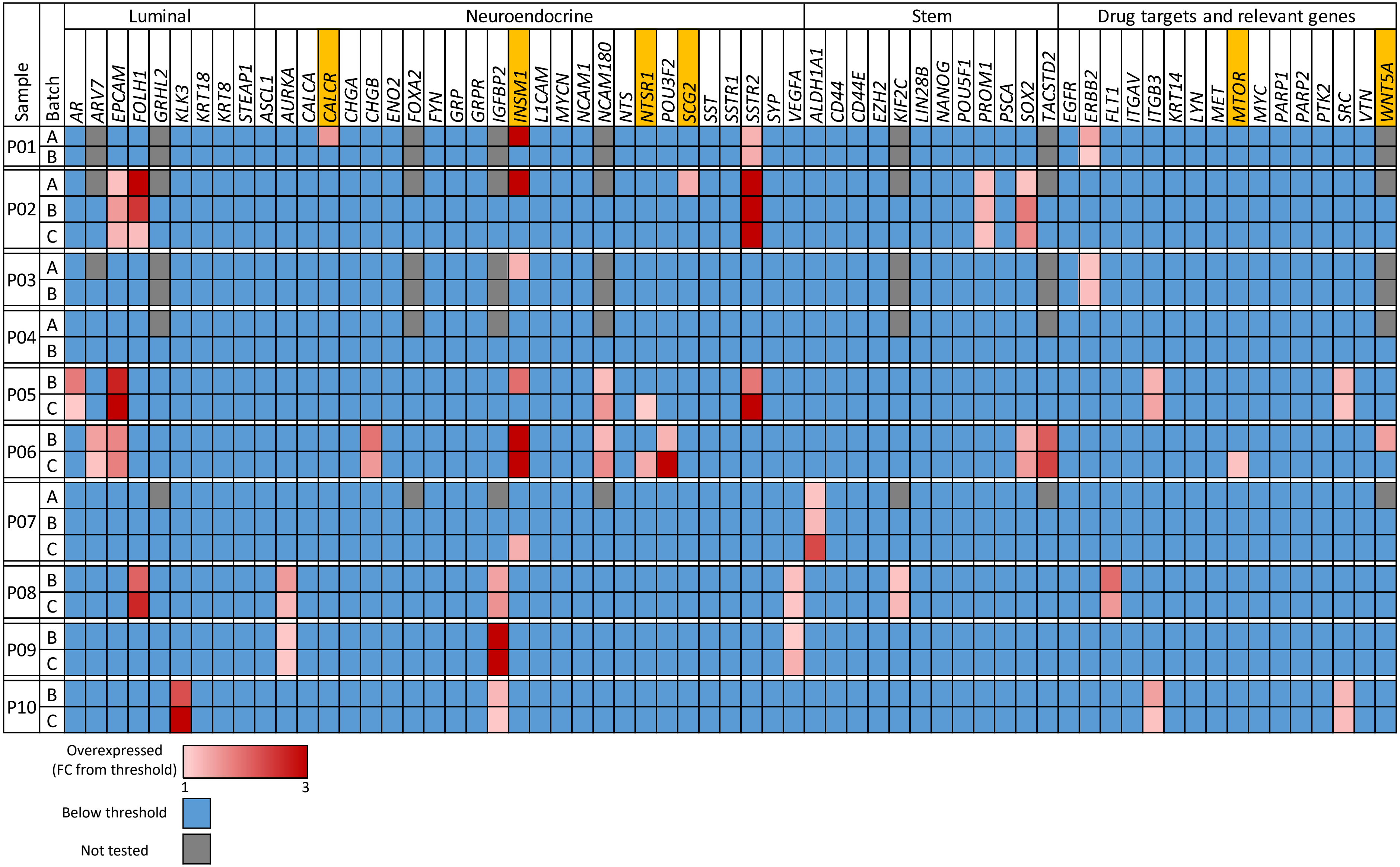

Figure S3: Reproducibility of gene expression in patients' blood RNA.

Results are presented as a heatmap, where gene overexpression is presented as a fold change from the threshold in a gradient of pink to red. Experimental batches are represented as A, B, and C. For genes with no expression in controls (e.g. *KLK3* and *FOLH1*), the gradient represents the fold change from the lowest positive signal in patients. Blue represents expression below overexpression threshold. Grey represents genes not tested in the batch. The six genes in orange showed inconsistent results and were excluded from further analyses.
